# Bioengineering methods for vascularizing organoids

**DOI:** 10.1016/j.crmeth.2024.100779

**Published:** 2024-05-16

**Authors:** Peter N. Nwokoye, Oscar J. Abilez

**Affiliations:** 1 Department of Medicine, Stanford University School of Medicine, Stanford, CA 94305, USA; 2 Department of Cardiothoracic Surgery, Stanford University, Stanford, CA 94305, USA; 3 Division of Pediatric CT Surgery, Stanford University, Stanford, CA 94305, USA; 4 Cardiovascular Institute, Stanford University, Stanford, CA 94305, USA; 5 Maternal and Child Health Research Institute, Stanford University, Stanford, CA 94305, USA; 6 Bio-X Program, Stanford University, Stanford, CA 94305, USA

**Keywords:** bioengineering methods, vascularization, organoids, organoid-on-a-chip, human pluripotent stem cells

## Abstract

Organoids, self-organizing three-dimensional (3D) structures derived from stem cells, offer unique advantages for studying organ development, modeling diseases, and screening potential therapeutics. However, their translational potential and ability to mimic complex *in vivo* functions are often hindered by the lack of an integrated vascular network. To address this critical limitation, bioengineering strategies are rapidly advancing to enable efficient vascularization of organoids. These methods encompass co-culturing organoids with various vascular cell types, co-culturing lineage-specific organoids with vascular organoids, co-differentiating stem cells into organ-specific and vascular lineages, using organoid-on-a-chip technology to integrate perfusable vasculature within organoids, and using 3D bioprinting to also create perfusable organoids. This review explores the field of organoid vascularization, examining the biological principles that inform bioengineering approaches. Additionally, this review envisions how the converging disciplines of stem cell biology, biomaterials, and advanced fabrication technologies will propel the creation of increasingly sophisticated organoid models, ultimately accelerating biomedical discoveries and innovations.

## Teaser

In this review, Nwokoye and Abilez discuss pioneering bioengineering strategies for organoid vascularization, such as co-culturing with vascular cells and utilizing organoid-on-a-chip technologies, and highlight the significant potential of these techniques to improve organoid functionality and maturation, thereby accelerating progress in biomedical research and applications.

## Introduction

Organoids are three-dimensional (3D) and self-organizing structures capable of recapitulating critical aspects of *in vivo* organ complexities and functionalities under specific physical, microarchitectural, and signaling cues.^1^^,^^2^ Representing a revolutionary advancement in modern biomedical research, 3D organoids hold unparalleled translational potential in regenerative medicine, disease modeling, and drug testing within human-specific contexts. While traditional monolayer two-dimensional (2D) culture systems have elucidated foundational cellular processes,^3^ they often fail to replicate the intricate architectural features and diverse cell-cell interactions crucial for a comprehensive understanding of native tissues.^4^^,^^5^

As the field strives to develop physiologically relevant organoids for regenerative medicine and translational research, the demand for bioengineered functional vasculature becomes increasingly apparent.^1^ In native tissues, the vasculature functions in nutrient supply and regulates tissue homeostasis, regeneration, and organ functionality.^6^ Similarly, vascularization is crucial to organoid cultures as it facilitates oxygen delivery, nutrient transport, and metabolic waste removal, all vital for organoid viability.^7^ While diffusion remains efficient for smaller organoids, it is inadequate beyond a specific size. The diffusion limit of oxygen and nutrients in mammalian tissues is approximately 100–200 μm, imposing significant physical constraints on the growth and longevity of larger constructs.^8^ In the absence of a functional vasculature, core regions of larger organoids often suffer from hypoxia and reduced nutrient access, resulting in necrosis and impaired functionality.^9^ Thus, it is vital to engineer intricate vascular networks within these 3D organoid structures to fully realize their therapeutic use.

**Figure 1:**
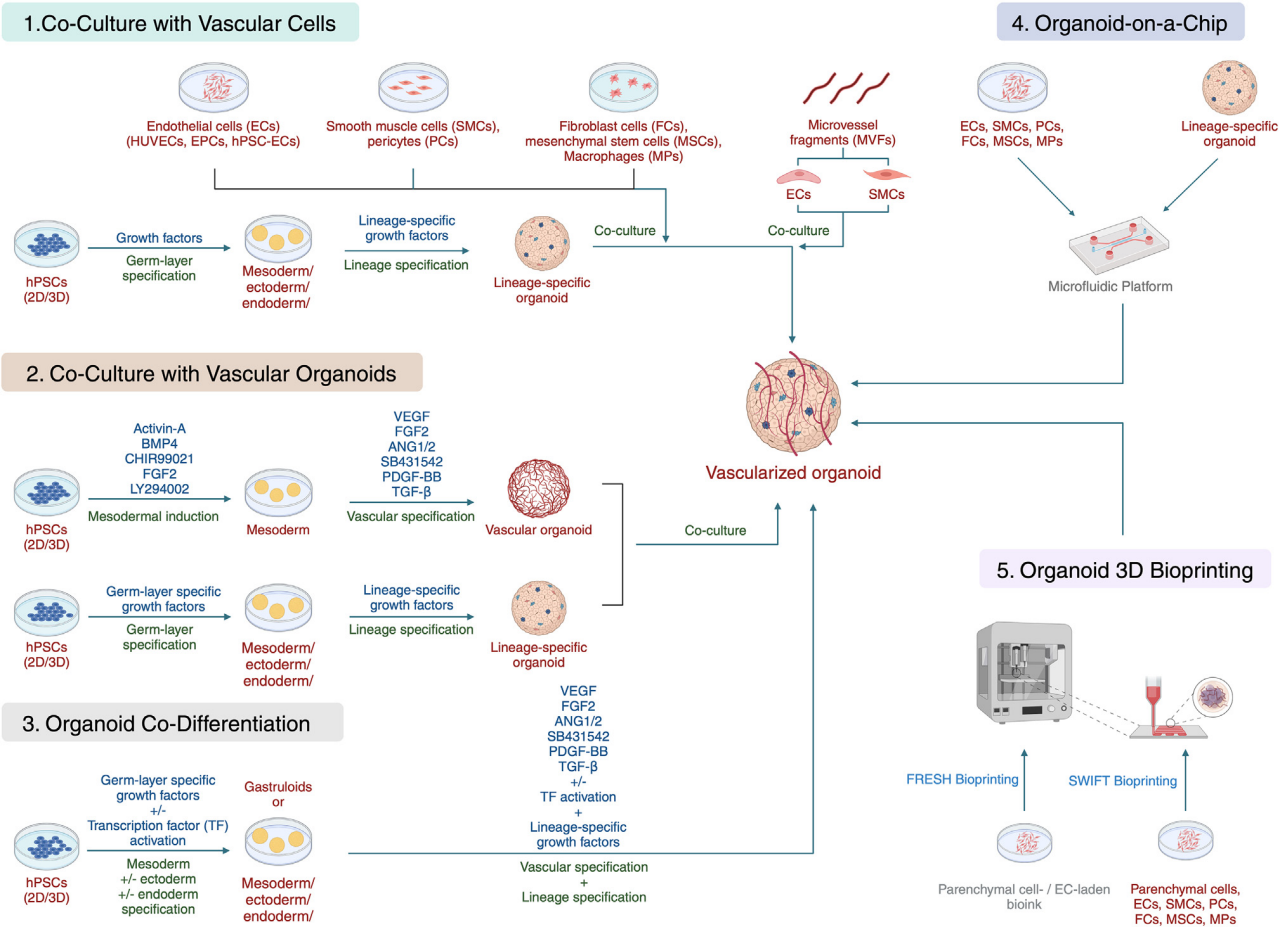
Schematic of the bioengineering methods for vascularizing organoids Organoids can be vascularized through co-culture with vascular cells, co-culture with vascular organoids, organoid co-differentiation, OOC platforms, and organoid 3D bioprinting. Created with BioRender.com.

Bioengineering strategies offer promising solutions to address the challenge of organoid vascularization (Figure 1) and include the following:

In co-culture with vascular cells, the introduction of endothelial cells (ECs) and supporting cell types (such as pericytes and fibroblasts) encourages the self-assembly of vessel-like structures within the organoid.

In co-culture with vascular organoids, the strategic combination of lineage-specific organoids with pre-formed vascular organoids facilitates integration and promotes vascularization.

In organoid co-differentiation, simultaneous differentiation of stem cells into both organ-specific and vascular lineages allows for integrated development of the organoid and its supporting vasculature.

In organoid-on-a-chip (OOC), integration of organoids into microfluidic devices enables the creation of perfused, vascularized systems, offering enhanced physiological relevance.

In organoid 3D bioprinting, the precise deposition of cells, biomaterials, and sacrificial inks permits the fabrication of organoids with pre-defined vascular channels, providing greater control over vascular architecture.

In this review, we delve into the rapidly evolving field of organoid vascularization. We begin by highlighting fundamental principles of vascular biology, emphasizing the complex interplay between stem cell niches and developing vascular networks, as well as the influence of biophysical cues such as fluid shear stress and extracellular matrix (ECM) composition. We then offer a critical evaluation of the specific vascularization strategies outlined above, discussing their strengths, limitations, current challenges, and potential applications in areas such as disease modeling and regenerative therapies. Finally, we provide a forward-looking perspective on the field, exploring how the convergence of stem cell biology, biomaterials science, and advanced microfabrication technologies promises to redefine the landscape of organoid research, propelling us toward the creation of organoid models with unprecedented sophistication and physiological relevance.

## Microvessels in vascular biology

Arterioles, capillaries, and venules are critical components of the vascular system, each with distinct structures and functions.^10^ Arterioles, the smallest arteries, range from 10 to 100 μm in diameter and feature a three-layered structure: tunica intima (innermost), tunica media, and tunica externa (outermost), allowing precise blood flow regulation.^11^ Their walls comprise a single layer of ECs that manage vascular tone and permeability, alongside mediating inflammation and coagulation. The smooth muscle cells in the tunica media enable arterioles to adjust to blood flow through vasoconstriction or vasodilation, while the tunica externa provides structural support. Blood flow control in arterioles responds to various stimuli, including neural and hormonal signals, and local metabolites, with nitric oxide from ECs facilitating vasodilation. Arteriolar dysfunction is linked to numerous vascular pathologies, such as hypertension, due to increased resistance, ischemic and hemorrhagic strokes from blood flow changes, peripheral artery disease causing limb pain and necrosis, and diabetic complications such as retinopathy, nephropathy, and neuropathy due to blood flow impairments.

Capillaries, with diameters of 5–10 μm, are composed of a single layer of ECs and a basement membrane, serving as primary sites for fluid and solute exchange between blood and tissues.^12^ Their structure allows for low-velocity, high-surface-area flow, optimizing the exchange of gases, nutrients, and wastes. Capillaries vary structurally as continuous, fenestrated, or sinusoidal types, reflecting tissue demands and their permeability.^13^ Continuous capillaries, found in muscles, skin, and the CNS, feature tight endothelial barriers regulated by tight-junction proteins, crucial for maintaining the blood-brain barrier. Fenestrated capillaries have pores for larger molecule passage and are located in the kidneys, intestines, and endocrine glands. Sinusoidal capillaries, the most permeable, are present in the liver, spleen, and bone marrow, where they facilitate the transfer of larger substances. Capillary dysfunction contributes to various pathologies. Inadequate capillary density or growth can lead to coronary heart disease and myocardial ischemia, while capillary rarefaction is associated with heart failure. Neurological impacts include contributions to ischemic stroke and Alzheimer’s disease through disrupted blood flow or blood-brain barrier integrity. Respiratory conditions such as chronic obstructive pulmonary disease (COPD) and pulmonary hypertension are linked to capillary density reduction and dysfunctional growth, respectively.

Venules are the microvessels linking capillaries to veins. They function in returning deoxygenated blood to the heart. They comprise three layers: the endothelial layer for fluid and solute exchange and blood component interaction, the basement membrane for structural support and regulation, and pericytes for vascular integrity and blood flow regulation. ECs are identified by molecular markers such as PECAM-1, VE-cadherin, ICAM-1, and vascular endothelial growth factor receptor (VEGFR).^14^ Venules are characterized by low blood pressure and velocity, facilitating white blood cell migration into tissues. Venules are categorized into post-capillary and muscular types. Post-capillary venules, which are 10–30 μm in diameter, have minimal smooth muscle and highly permeable ECs, enabling efficient exchange with surrounding tissues. Muscular venules, larger at 30–100 μm, contain more smooth muscle layers, allowing them to adjust blood flow and pressure in response to stimuli.^15^^,^^16^ Venular dysfunction is implicated in numerous vascular diseases, including inflammation, cancer metastasis, and neurovascular disorders.

## Stem cell niches and vascularization

Stem cell niches constitute highly specialized microenvironments where intricate crosstalk between stem cells, their progeny, and neighboring vasculature orchestrates fundamental biological processes. This includes the dynamic interplay of vasculogenesis (*de novo* vessel formation) and angiogenesis (sprouting from existing vessels), processes crucial for mediating stem cell quiescence, self-renewal, and lineage commitment. Organoids, derived from stem or progenitor cells within these niches, offer unprecedented opportunities to dissect these mechanisms, holding transformative potential for regenerative medicine.^7^ However, replicating the intricate vascular networks characteristic of *in vivo* niches remains a central challenge in organoid systems. Elucidating the spatiotemporal dynamics governing stem cell-vasculature interactions, particularly those driving vasculogenesis and angiogenesis, is paramount for advancing the physiological relevance of organoid models.

Vascular development is orchestrated by a complex interplay of molecular factors and signaling pathways, facilitated by the intrinsic ability of ECs to self-organize into tubular structures when exposed to appropriate cues.^17^^,^^18^ Generating vascular networks in organoids typically begins with mesoderm induction in aggregates of human pluripotent stem cells (hPSCs), often achieved through a combination of Activin-A, bone morphogenetic protein 4 (BMP-4), CHIR99021 (a Wnt pathway inhibitor), fibroblast growth factor (FGF)-2, and LY294002.^19^^,^^20^ Subsequent vascular induction and angiogenesis rely on several additional key mediators.

VEGF-A is arguably the most potent driver of both processes, interacting with its receptors (VEGFR1 and VEGFR2) on ECs. FGF-2 further supports EC proliferation and differentiation.^21^ The angiopoietin-Tie2 signaling axis is critical for vessel maturation and stabilization.^22^ Members of the transforming growth factor (TGF)-β superfamily, Notch ligands, and both canonical and non-canonical Wnt pathways also play essential roles in vascular development. The Ephrin/Eph receptor system is crucial for arterial/venous specification,^23^ and Wnt signaling is involved in diverse aspects of vascular patterning.^24^^,^^25^ Finally, platelet-derived growth factor (PDGF)-β signaling is instrumental in the recruitment of pericytes and smooth muscle cells to ECs, crucial for vessel stability.^19^

The specificity in dosage and timing of these factors, particularly VEGF-A, significantly shapes vascular patterning within organoids.^26^ Furthermore, as organoids progress through developmental stages, their evolving metabolic needs can alter the optimal VEGF-A dynamics, potentially affecting vascularization efficiency. The low oxygen tension at the core of large organoid constructs triggers endogenous VEGF-A production,^2^ introducing batch variability. Hence, engineering optimal vascularization requires iterative experimentations, factoring in the manifold interactions of VEGF-A within the angiogenic milieu alongside the unique requirements of the specific organoid system.

**Figure 2:**
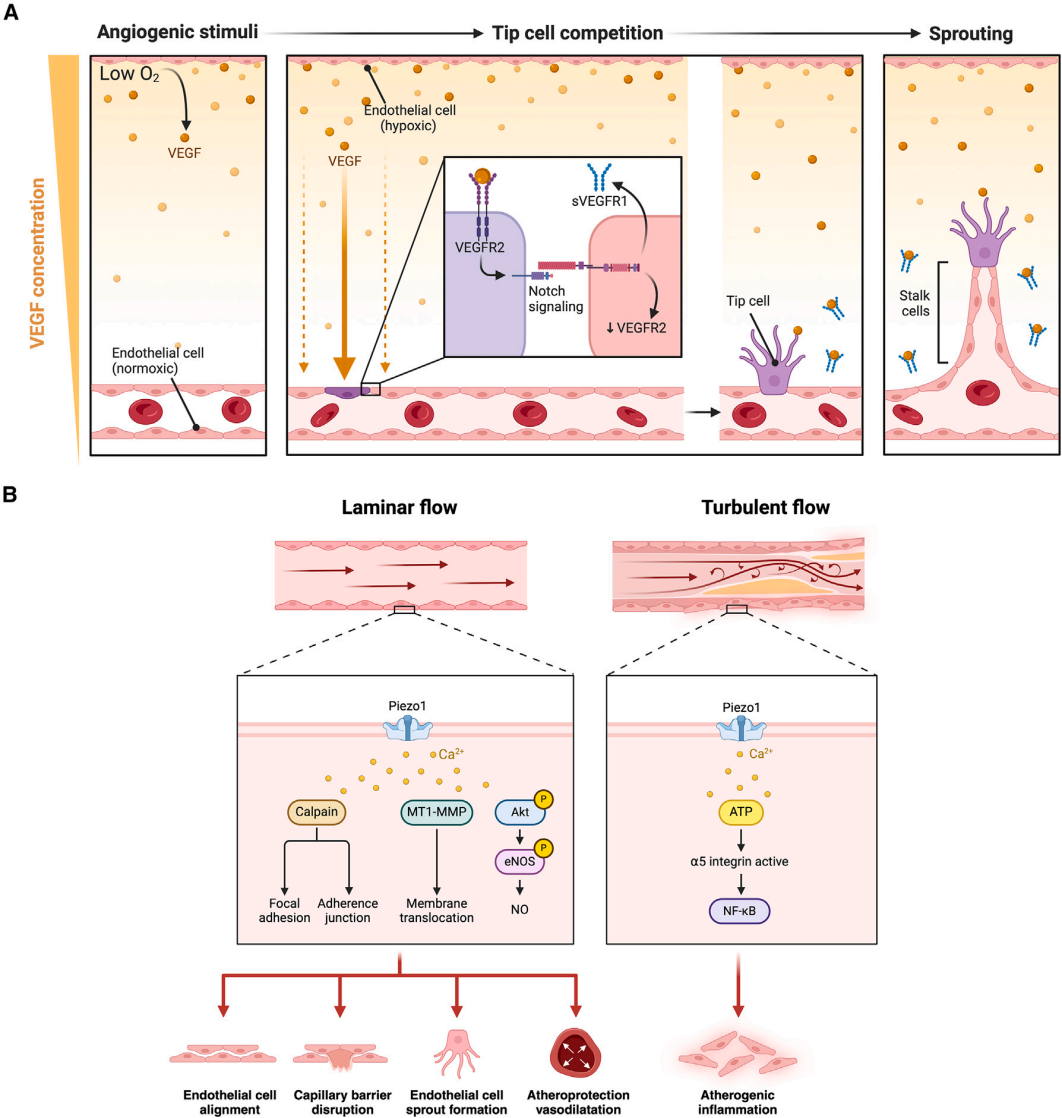
VEGF gradient-driven angiogenesis and Piezo1-mediated EC signaling (A) VEGF gradient-driven angiogenesis in response to hypoxia. Angiogenic stimuli: ECs lining the blood vessels are exposed to gradients of VEGF, with concentrations inversely proportional to oxygen tension in the tissue. Tip cell competition: in response to the angiogenic gradient, VEGFR2 is expressed on one cell, stimulating Notch signaling in the neighboring cell. This intercellular communication results in the downregulation of VEGFR2 and upregulation of sVEGFR1, which binds to and sequesters free VEGF. The communication between the adjacent tip cells determines which cell becomes the tip cell and which remains a stalk cell. Sprouting: the tip cell leads the sprouting angiogenesis, extending over the elongating stalk cells toward the hypoxic region. Reprinted from “The Process of Sprouting Angiogenesis in a Healthy Blood Vessel,” by BioRender.com (2024). (B) Piezo1-mediated EC signaling in response to flow conditions. Under laminar flow (left), Piezo1 channel activation results in calcium influx, which initiates a cascade of downstream effectors, including calpain, MT1-MMP, and Akt. These pathways effect several physiologic changes in the ECs. In turbulent conditions (right), Piezo1 activation results in activation of α5-integrin, promoting atherogenic inflammation of vessels over time. Adapted from “Piezo1 Endothelium Signaling,” by BioRender.com (2024). Retrieved from https://app.biorender.com/biorender-templates.

Under *in vivo* conditions, vascular growth and branching are exquisitely guided by gradients of angiogenic signals, a dynamic interplay difficult to replicate within organoids. As illustrated in Figure 2A, hypoxia triggers a VEGF gradient, with nearby cells secreting VEGF in concentrations inversely proportional to oxygen tension. ECs, responding to VEGF via VEGFR2, engage in Notch-mediated lateral inhibition. This process designates tip cells (high VEGFR2) to lead the angiogenic sprout along the VEGF gradient, while stalk cells (high sVEGFR1) elongate the vessel.

To fully realize the potential of vascularized organoids in deciphering stem cell biology and advancing regenerative therapies, overcoming the challenges in replicating complex vascular networks remains paramount. Future studies must meticulously dissect the intricate interplay between signaling gradients and the spatiotemporal dynamics that distinguish vasculogenesis from angiogenesis. This understanding will inform the development of sophisticated organoid models where exogenous factor supplementation aligns precisely with the organoids’ evolving metabolic and signaling requirements. Moreover, achieving spatiotemporal control over key angiogenic pathways will enable the engineering of physiologically relevant vascular structures. Such advancements will empower researchers to unravel the complex crosstalk between stem cells and their vascular niche, ultimately accelerating breakthroughs in disease modeling and regenerative therapies.

## Influence of fluid shear stress on vascularization

In vascular biology, the interaction of ECs with their physical environment is critical. These cells are intrinsically mechanosensitive, allowing them to respond to fluid shear stresses, a key aspect of the *in vivo* hemodynamic environment.^27^ This shear stress, the result of blood flow, plays an important role in regulating vascular development and remodeling *in vitro* and *in vivo*.^27^

During embryonic development, the formation and specification of the arteries and veins depend in part on the hemodynamic forces on the developing vessels. Specifically, the shear stress gradients influence EC gene expression, structure, and function.^27^ Arterial and venous specifications are guided by high and low shear stresses, respectively. High shear stress results in the upregulation of EphrinB2, an arterial marker, which is upregulated by 2-fold at a shear stress of 20 dyne/cm^2^, while lower shear stress induces the expression of EphrinB4, a venous marker, which is upregulated by 3-fold at a shear stress of 5 dyne/cm^2^.^28^ Moreover, shear stress can influence the localization and activation of mechanosensory complexes, such as PECAM-1 and VE-cadherin, which maintain junctional integrity in ECs.^29^

A thorough understanding of the interplay between *in vivo* shear stress and vascular development is central to engineering functional and hierarchical organoid vessels. As discussed in subsequent sections, OOC and microfluidic platforms enable controlled fluidic microenvironments, improve the biomimicry of *in vivo* shear stresses, and guide the formation of physiologically relevant vessels. OOC platforms can be used to study the effects of shear stress on vascularization in a more controlled and relevant setting compared to traditional cell culture methods. By incorporating microfluidic channels and applying controlled fluid flow, these platforms can generate physiological shear stresses that promote the formation of functional and hierarchical vascular networks within organoids. In a seminal study, Huh et al. developed a biomimetic lung-on-a-chip to recreate the alveolar-capillary interface of the human lung.^30^ The model recapitulated the lung parenchyma and incorporated endothelial channels to investigate blood-air barrier dynamics and mechanosensitive pulmonary responses to nanoparticulates. The use of controlled cyclic mechanical strain with a frequency of 0.2 Hz and a magnitude of 10% strain was shown to induce the alignment of ECs.^30^

Several molecular signals and mechanotransduction pathways involving ECs have been identified,^31^ although the precise roles and temporal activation patterns of the critical drivers are still under active research. One widely recognized mechanotransduction pathway involves Piezo1, a non-selective cation channel located on the luminal side of ECs. This channel is sensitive to varying flow conditions, prompting calcium influx into ECs and subsequently initiating a cascade of downstream responses.^32^ Figure 2B shows Piezo1-mediated EC responses under laminar and turbulent flows. Under laminar flow conditions, the activation of the channel and subsequent calcium influx can lead to structural adaptations in the vessel, promoting physiological vascular remodeling. Conversely, under turbulent flow, the downstream response may contribute to atherogenic inflammation.

In engineering vascularized organoids, it is essential to understand the mechanosensory role of Piezo1, particularly in vascular remodeling and ECM interactions. Piezo1 signaling has the potential to influence endothelial-to-mesenchymal transition (EndoMT),^33^ a process implicated in vascular remodeling and pathological conditions. Moreover, the interaction of Piezo1-mediated signaling in ECs with perivascular and stromal cells warrants further investigation. Gaining insight into these interactions could be crucial for engineering lumenized and hierarchical vessels within organoids and designing sophisticated microfluidic devices. Furthermore, research suggests that the surrounding ECM scaffold significantly influences Piezo1-mediated mechanosensation in ECs. Lai et al. demonstrated that Piezo1 sensitivity to shear stress is regulated by integrin-mediated interactions with the ECM.^34^ Specifically, fibronectin is linked to the sensitization of Piezo1 response under high shear stress conditions (via α5β1- and αvβ3-integrins), while laminin and collagen types I and IV promote Piezo1 sensitivity under low shear stress conditions (via αvβ3- and αvβ5-integrins).

Engineering physiologically relevant vascularization within organoids demands a comprehensive understanding of the mechanobiological environment governing EC behavior. While Piezo1’s role in shear stress sensing is well established,^32^^,^^35^ a diverse network of mechanosensory proteins contributes to vascular development and remodeling. This network includes ion channels of the TRP family and potassium channels, which modulate flow-mediated dilation. Moreover, junctional complexes involving VE-cadherin and PECAM-1 transduce shear stress signals, affecting endothelial barrier function.^29^ Integrins, the primary mediators of cell-ECM interactions, are crucial in transmitting mechanical cues and influencing EC responses.^36^ Krüppel-like factors 2 and 4 (KLF2 and KLF4) are critical transcription factors that directly respond to flow-induced shear stress in ECs.^37^ This mechanosensitive response enables these factors to govern endothelial gene expression, promoting anti-inflammatory, anti-thrombotic, and vasoprotective states. KLF2 and KLF4 are essential for adapting the vasculature to fluctuations in blood flow patterns, ensuring homeostasis and preventing pathologies such as atherosclerosis.^37^

Understanding the interplay among these mechanosensors, shear stress gradients, and ECM composition is essential for engineering vascularized organoids that accurately recapitulate *in vivo* vascular behavior. This knowledge will empower the precise manipulation of organoid microenvironments to control vessel formation, branching, and permeability. Furthermore, detailed exploration of these mechanosensory pathways may reveal novel therapeutic targets for modulating vascular development and dysfunction within organoid models of disease.

## Scaffolds for engineering vascularization

**Figure 3:**
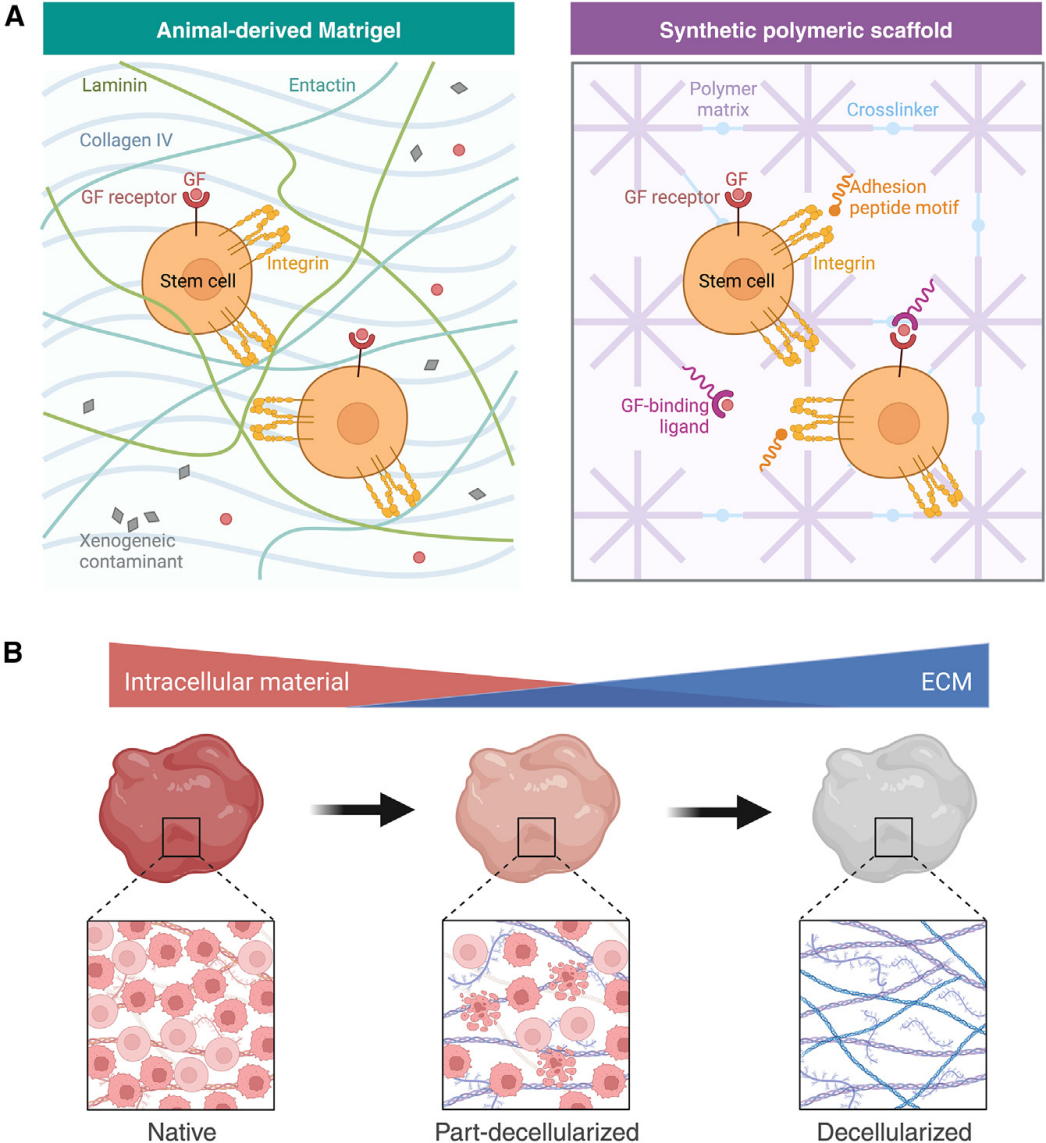
Various scaffolds for engineering vascularization (A) Comparison of animal-derived (left) and synthetic (right) scaffolding materials for organoid engineering. Animal-derived Matrigel is characterized by complex and variable matrix components in undefined ratios. Moreover, the presence of xenogeneic contaminants and proteins can result in undesirable effects and batch variability. The synthetic polymeric scaffold (right) has components in well-defined ratios with highly tunable physicochemical properties, offering a controlled cellular response. (B) Decellularization process of native tissue. The progression from left to right illustrates the transition from a native tissue, characterized by abundant cellular components, to a partially decellularized state with reduced intracellular material, and subsequently to a fully decellularized matrix, rich in extracellular matrix (ECM) components. The intricate ECM architecture is preserved for use in organoid vascularization. Reprinted from “Comparison of Matrigel and Synthetic Scaffolds” and “The Decellularization Effect on the Extracellular Matrix (ECM)” by BioRender.com (2024). Retrieved from https://app.biorender.com/biorender-templates.

ECs embedded within hydrogel scaffolds exhibit exquisite sensitivity to the biophysical properties of their microenvironment.^38^ This mechanotransduction—the conversion of mechanical stimuli into biochemical signals and vice versa—plays a critical role in vascular development and function. For endothelial sprouting and the development of perfusable vessels, a precise balance between scaffold adhesiveness and controlled degradation is necessary to promote EC invasion and matrix remodeling.^39^ Scaffold characteristics, including stiffness and elasticity, significantly modulate EC behavior. Studies demonstrate that softer scaffolds promote endothelial sprouting and microvascular network formation.^40^ Moreover, variations in stiffness elicit diverse EC responses with implications for vascularized organoid engineering. To better understand and harness these mechanotransductive processes, animal and naturally derived scaffolds (Figure 3A, left), including Matrigel, alginate, collagen, and fibrin, possess inherent biocompatibility that makes them invaluable scaffolds for investigating angiogenesis, cell proliferation, and differentiation.

Collagen, the most abundant protein of mammalian ECM, provides mechanical support through its fibrillar structure. This facilitates cellular adhesion, proliferation, and differentiation—processes essential for neovascularization.^41^^,^^42^ Collagen-based hydrogels, by virtue of their biocompatibility and bioactive nature, effectively recapitulate aspects of the vascular ECM. Gelatin, a denatured collagen derivative, retains many of collagen’s cell-interactive properties.^43^ Gelatin methacryloyl (GelMA), a photocrosslinkable functionalized gelatin, has become widely used in vascular tissue and organoid engineering.^44^^,^^45^ Its photopolymerizable nature allows for precise modulation of mechanical properties and degradation kinetics, offering tailored control for the engineering of blood vessels. In hard-tissue applications such as bone, more rigid hydrogels—modified gelatins or collagen composites—provide both the structural support conducive to bone formation and the capacity to promote vascularization.^46^ Fibrin is a key ECM component that plays a fundamental role in wound healing and blood vessel formation. Fibrin hydrogels simulate the natural wound matrix, supporting EC migration and tube formation.^47^^,^^48^ Alginate and hyaluronic acid, polysaccharides prevalent in the ECM, offer distinct advantages for promoting tissue vascularization. Alginate has gentle gelation conditions and is thus recognized for its ability to encapsulate cells with minimal cytotoxicity.^49^ Hyaluronic acid hydrogels have been instrumental in elucidating the dynamic interplay between ECs and the vascular basement membrane.^50^

While naturally derived hydrogels offer biomimicry, their mechanical properties can be less easily controlled. In contrast, synthetic hydrogels represent a paradigm shift in tissue engineering, offering tailorable biomechanical platforms that precisely recapitulate the dynamic microenvironments of native tissues (Figure 3A, right). Polyethylene glycol (PEG)-based hydrogels exhibit favorable properties for tissue vascularization, including hydrophilicity, biocompatibility, and resistance to nonspecific protein and cell adhesion.^51^ The ability to incorporate bioactive moieties (e.g., VEGF, arginylglycylaspartic [RGD] acid peptide) enhances EC behavior and supports multi-cellular co-culture systems.^52^^,^^53^ Other synthetic polymers, such as polylactic acid (PLA), poly(lactic-co-glycolic acid) (PGLA), and polycaprolactone (PCL), provide additional options for tailored applications.^54^

To study microvascular formation and vascular remodeling, researchers often turn to PEG-based hydrogels since their rheological properties are highly tunable. Friend et al. demonstrated the acceleration of vessel growth and cell-mediated stiffening using adjustable PEG-norbornene (PEGNB) hydrogel.^55^ Notably, researchers recently devised a tissue-mimetic synthetic ECM to investigate the interactions between epithelial and stromal cells in the endometrium during menstruation—a process intrinsically characterized by vascular remodeling. Importantly, by tuning its physicochemical properties to mimic *in vivo* conditions, this synthetic ECM can serve as a model to investigate normal endometrial physiology or pathologies.^56^ Despite these recent strides, a notable challenge remains: synthetic ECMs might not fully mimic the intricate biosignaling cues essential for remodeling across diverse tissues.

To further enhance control and bioactivity, researchers have also turned to hybrid bioinks, which leverage the strengths of both natural and synthetic materials. These composite systems offer a range of advantages for vascularizing organoids. For instance, combining decellularized ECM (dECM) with photocrosslinkable synthetic polymers such as PEG-methacrylate enables precise tuning of stiffness and degradation profiles while maintaining the biochemical complexity of the native tissue microenvironment.^57^ Additionally, biofunctionalization of synthetic components with cell-adhesive motifs (e.g., RGD peptides) or pro-angiogenic growth factors (e.g., VEGF) provides targeted support for EC function and vascular network formation.^58^ However, a key challenge lies in balancing biocompatibility with the potential for reduced cell responsiveness in highly synthetic environments. Ongoing research focuses on developing advanced hybrid bioinks, including interpenetrating networks (IPNs) of natural and synthetic polymers, and utilizing bio-orthogonal “click” chemistries.^59^ These techniques enable the temporal and spatial control of ECM properties and bioactive factor presentation, allowing for dynamic guidance of vascularization processes within organoids. Such advancements could ultimately lead to more complex and physiologically relevant vascular architectures within organoids.

Researchers have developed stimuli-responsive hydrogels that dynamically interact with their microenvironment, more accurately replicating the complexity of both physiological and pathological processes.^60^ These “smart” hydrogels respond to cues such as temperature, pH, light, and protein, offering unprecedented control over growth factor release and closely mimicking the dynamic nature of the native ECM.^61^ This positions them to transform tissue vascularization strategies. These hydrogels model the cellular environment by replicating the physical, mechanical, and biological characteristics of natural tissues, promoting robust cell growth, 3D structural support, and targeted delivery of bioactive agents. Notably, the photopolymerizable hydrogel, poly(vinyl alcohol) (PVA) methylacrylate enables high-resolution spatial patterning, facilitating the fabrication of intricately vascularized tissues.^62^^,^^63^ Furthermore, smart hydrogels have demonstrated potential in bone repair applications. Yang et al. developed an enzyme-sensitive hydrogel microsphere that releasers bone marrow mesenchymal stromal cell-derived exosomes (BMSC-Exos) specifically in response to neovessel formation during bone healing. This MMP1-responsive system promotes stem cell migration and osteodifferentiation within the newly vascularized areas.^64^ For a broader perspective on the diverse applications of smart hydrogels in tissue engineering, refer to El-Husseiny et al.^65^

Decellularized extracellular matrices (dECMs), derived from native tissues, offer a promising avenue for engineering vascularized organoids. By removing cellular components through a decellularization process (involving detergents, enzymatic treatments, and physical disruption), dECMs retain tissue-specific biochemical cues and structural architecture (Figure 3B).^66^^,^^67^ This biomimetic scaffold provides crucial mechanical support and a rich signaling environment for embedded cells and sprouting vessels.^68^ Advantages of dECMs include their inherent biocompatibility, the presence of vascular-relevant growth factors, and the potential for fine-tuning their mechanical properties.^69^ However, challenges persist, such as batch-to-batch variability, the risk of incomplete decellularization, and the need to optimize re-endothelialization strategies. Emerging solutions include developing standardized decellularization protocols, creating biohybrid dECM bioinks with synthetic components for enhanced control, and utilizing sophisticated microfluidic platforms to guide vascularization within dECM scaffolds.^70^ By addressing these challenges and harnessing the potential of dECMs, researchers can engineer organoids with more physiologically relevant and functional vascular networks.

## hPSCs for creating vascularized organoids

hPSCs, including human embryonic stem cells (hESCs) and human induced pluripotent stem cells (hiPSCs), offer unparalleled potential for regenerative medicine and disease modeling. The seminal discovery of somatic cell reprogramming into hiPSCs via Yamanaka factors (OCT3/4, SOX2, KLF4, c-MYC) has enabled the creation of patient-specific cell lines, circumventing ethical concerns associated with hESCs and facilitating personalized therapies.^71^ Given their capacity to differentiate into virtually any cell type, including the ECs and mural cells (smooth muscle cells and pericytes) that constitute blood vessels, hPSCs represent a powerful resource for engineering complex vascularized tissues and organoids.

Two principal strategies drive hPSC differentiation: embryoid body (EB) (3D) directed differentiation and monolayer (2D) directed differentiation.^72^^,^^73^ EB formation, a 3D aggregation technique, simulates aspects of early embryogenesis through cell-cell interactions, fostering differentiation into multiple lineages, including vascular cells.^72^^,^^74^ Monolayer directed differentiation provides greater convenience in day-to-day cell culture techniques as media changes with EBs poses the chance of aspirating free-floating colonies. Both 3D and 2D approaches use factors and small molecules such as Activin-A, BMP-4, CHIR99021, FGF-2, LY294002, VEGF-A, angiopoietins 1 and 2, SB431542, PDGF-BB, and TGF-β to promote mesoderm and subsequent vascular lineage specification.^75^ For vascularization studies, EBs can provide a physiologically relevant model, although early differentiation protocols generated vascular cells with low efficiency (1%–5%). Monolayer methods, while often more efficient (5%–20%), favor mechanistic investigations of EC differentiation but historically rely on undefined media.^76^ Advancements over the past decade have defined chemically defined protocols for both approaches, enhancing reproducibility, scalability, and identification of key signaling pathways underlying hPSC-derived vascular cell differentiation, including ECs (CD31^+^, VE-Cadherin^+^), smooth muscle cells (SMCs) (α-SMA^+^, SM22α^+^), and pericytes (PDGFR-β^+^, NG2^+^),^77^^,^^78^^,^^79^^,^^80^^,^^81^ as further discussed below.

In 2012, Dar et al. unveiled one of the earliest protocols for generating vasculogenic pericytes from hPSCs, expanding the repertoire of hPSC-derived vascular cell types.^82^ They identified a novel population of cells positive for pericyte markers (PDGFR-β, NG2) but not for α-SMA, displaying characteristics of mesenchymal stem cells. The absence of α-SMA suggests that these hPSC pericytes are immature,^83^ which may limit their physiological relevance. While this immaturity may constrain studies focusing on pericyte-mediated vessel stabilization, these hPSC-derived pericytes demonstrated integration with host vasculature and enhanced recovery in a murine limb ischemia model. Crucially, Dar et al. revealed the multipotent nature of hPSC-derived pericytes, highlighting their capacity for osteogenic, chondrogenic, adipogenic, and myogenic differentiation. This multipotency underscores their relevance in vascular remodeling, tissue regeneration, and the study of vascular pathologies.

The last decade has witnessed remarkable progress in tailoring hPSC differentiation toward vascular and perivascular cell lineages. Seminal studies by Giacomelli et al. and Orlova et al. established the foundation for simultaneous hPSC differentiation into cardiomyocytes, ECs, and pericytes/mural cells.^81^^,^^84^ Their protocols leveraged precise modulation of Wnt, Activin/Nodal, VEGF, and TGF-β pathways via CHIR99021, Activin-A, BMP4, VEGF, and SB431542, guiding cells through mesodermal induction and subsequent cardiomyocyte and vascular specification. While the protocols employed chemically defined media and demonstrated cross-line reproducibility, efficiency variations (10%–30%) underscored the need for further refinement to ensure robust and consistent outcomes. Cheung et al. and Patsch et al. significantly advanced the field with a rapid, highly efficient protocol for differentiating hPSCs into ECs and SMCs.^76^^,^^85^ Their approaches involved initial GSK3 inhibition (via CHIR99021) and BMP4 treatment to drive mesodermal commitment, followed by lineage-specific exposure to VEGF-A (for ECs) or PDGF-BB (for SMCs). These strategies achieved differentiation efficiencies exceeding 80% within 6 days.

A breakthrough came with Wimmer et al., who pioneered self-assembling human vascular organoids from hiPSCs.^86^ These organoids exhibited complex architecture, with EC-lined branching capillaries expressing canonical markers (e.g., CD31, VE-cadherin) and integrated pericyte support. This model’s ability to recapitulate diabetic vasculopathy under hyperglycemic conditions highlights its potential for studying metabolic pathologies. However, limitations include a lack of interaction with crucial cell types (e.g., immune cells or fibroblasts), the inability to form more complex vessel types with arteriolar or venular characteristics, and potential batch-to-batch variability introduced by the collagen I-Matrigel matrix. Schmidt et al. recently presented an alternative model focused on early and late phases of human blood vessel development.^87^ Importantly, their approach employed a single VEGF pulse, omitting FGF-2 and forskolin—factors implicated in nascent vessel stabilization. The use of non-adhesive agarose-coated wells to generate 3D aggregates eliminated the Matrigel matrix. However, the model’s limited *in vitro* mural cell coverage suggests either incomplete differentiation or a specific focus on early sprouting-like vasculogenesis, potentially restricting its use for studies requiring vessel stabilization.

The capacity to generate ECs from hPSCs has enhanced our ability to study endothelial function and pathophysiology in a controlled setting. Patient-specific hPSCs hold particular promise for modeling genetic vascular disorders, offering “disease-in-a-dish” platforms that faithfully reflect disease genotypes and phenotypes. Exemplary work by Atchison et al. established a tissue-engineered model of Hutchinson-Gilford progeria syndrome, demonstrating the power of this approach.^88^ Moreover, hPSC-based vascular models are invaluable for investigating complex multifactorial disorders such as diabetes, enabling researchers to dissect the effects of hyperglycemia on microvascular dysfunction and explore therapeutic interventions.^86^

## Methods for engineering vascularized organoids

Engineering a physiologically relevant vascular architecture *in vitro*, with hierarchically branched networks and perfusable lumens, requires meticulous orchestration.^79^^,^^89^ Achieving this in the context of 3D organoids, which are innately complex due to their intricate cell-cell and cell-matrix interactions, further compounds the challenge. This task presents a unique multidisciplinary challenge that necessitates the integration of insights from developmental biology, material science, and bioengineering. This section delves into the various methods for engineering vascularized organoids, which include co-culture with vascular cells, co-culture with vascular organoids, organoid co-differentiation, OOC platforms, and organoid 3D bioprinting (Figure 1). We explore the myriad biological and technical challenges associated with *in vitro* vascularization, which have been roadblocks to fully realizing the translational potential of 3D organoids in regenerative medicine and beyond.

### Co-culture with vascular cells

To accurately recapitulate the complex cellular composition and signaling dynamics of the native vascular niche, researchers have shifted toward multi-cellular culture systems. Pioneering work by Levenberg et al. (2005) established the feasibility of co-culturing ECs with auxiliary cell lineages, including fibroblasts and skeletal muscle cells, to generate robustly vascularized 3D skeletal tissues.^90^ Building upon this foundation, Rouwkema et al. and Kyriakidou et al. successfully applied similar strategies within bone tissue engineering, further solidifying the potential of this multi-cellular paradigm for promoting physiologically relevant vascularization.^91^^,^^92^

The choice of EC source for vascularization experiments demands careful consideration. Commonly used options include human umbilical vein ECs (HUVECs), endothelial progenitor cells (EPCs), and hPSC-derived ECs (hPSC-ECs) (Table 1).^93^^,^^94^^,^^95^^,^^96^ HUVECs, due to their commercial availability and ease of culture, remain a prevalent choice for studying endothelial behavior and vascularizing *in vitro* models.^97^ EPCs, while crucial for postnatal endothelial repair and neovascularization, can be isolated from various sources (adult peripheral blood, umbilical cord blood, bone marrow).^98^ However, their broader adoption in vascular tissue engineering faces potential hurdles due to challenges in definitive EPC characterization, the need for highly selective identification markers, and the complexity of optimizing culture conditions to support robust microvessel formation. hPSC-ECs offer unparalleled advantages, such as versatility in differentiation into autologous ECs and perivascular cells.^84^

**Table 1:** Different cellular sources for generating ECs used for vascularizing organoids and tissue constructs

Cellular source	Markers	Advantages	Bioengineering challenges
HUVECs	*CD31* (*PECAM-1*), *CD144* (VE-Cadherin), vWF, eNOS	1. robust ability to form vessel-like structures *in vitro* and *in vivo* 2. ease of isolation and characterization 3. extensively studied in vascular research	1. source variability with differences in genetic and phenotypic characteristics between donors 2. limited *in vivo* functionality long term 3. prone to senescence during prolonged culturing 4. limited ability to form microvessels 5. HUVECs can overexpress inflammatory markers under certain conditions 6. ethical concern with supply in some parts of the world
EPCs	early EPC markers: *CD34*, *CD133*, *VEGFR-2* late EPC markers: *CD31* (*PECAM-1)*, *CD144* (VE-Cadherin), vWF, eNOS	1. inherent angiogenic capabilities 2. ability to home to ischemic sites 3. potential for rapid proliferation and maturation into ECs^1^	1. phenotypic heterogeneity due to lack of specific markers 2. finite proliferative potential in adult peripheral blood 3. challenges in achieving full maturity and function as mature ECs
hPSCs (hiPSCs/hESCs)	hPSC-ECs: *CD31* (*PECAM-1*), *CD144* (VE-Cadherin), vWF, eNOS loss of pluripotency markers: *LIN28*, *OCT4*, *NANOG*, *SOX2*, *SSEA-3*, *SSEA-4*, *TRA-1-60*, *TRA-1-81*, *UTF1*	1. patient-specific (hiPSCs) and autologous source for personalized medicine 2. versatility in differentiation into both ECs and perivascular cells 3. provides a renewable source of cells 4. can be precisely modified using the CRISPR-Cas9 system 5. physiologically relevant to model inflammatory conditions	1. achieving high and consistent yield of hPSC-ECs remains a challenge 2. risk of *in vivo* teratoma formation from residual undifferentiated cells 3. reprogramming somatic cells can result in genetic and epigenetic abnormalities 4. differentiation protocols are complex and resource intensive 5. hPSC-EC populations can be heterogeneous and contain off-target cells 6. some hPSC-ECs can be immunogenic

These different cellular sources are characterized by specific surface markers, and each has several advantages and bioengineering challenges associated with its use in vascularization systems.

Co-culturing ECs with strategically selected supporting cell types and pro-angiogenic factors (e.g., VEGF, FGF-2) drives self-organization into tissue-specific vascular structures. Recent advances incorporate mesodermal progenitor cells (MPCs), mesenchymal stem cells (MSCs), and macrophages for enhanced vascularization and maturation in organoid models.^99^ MPCs, with their mesodermal lineage potential, provide a vital source of pericytes and SMCs—mural cells essential for vessel integrity, contractility, and hemodynamic regulation.^100^ MSCs secrete a potent pro-angiogenic cocktail (including TGF-β, PDGF, and other cytokines) that stimulates EC sprouting and stabilizes nascent vessels.^101^^,^^102^ Macrophages play dynamic roles in angiogenesis, tissue remodeling, and innate immunity, ensuring these models reflect the critical immune-vascular interface and more closely mimic the *in vivo* microenvironment.^103^

MPCs, with their capacity to differentiate along endothelial, vascular smooth muscle, and potentially even hematopoietic lineages, are poised to facilitate organoid vascularization.^100^ This differentiation spectrum is critical for replicating the intricate cellular composition and architecture of native vascular networks. Dogan et al. harnessed the potential of hPSC-derived MPCs (hiMPCs) to bioprint complex vascular systems.^104^ By incorporating hiMPCs into a biocompatible alginate/collagen bioink, they observed the spontaneous formation of hierarchical vessels with multilayered walls—a hallmark of physiologic vasculogenesis. The utility of MPCs extends to the generation of functional organoids. Wörsdörfer et al. demonstrated how hiPSC-derived MPCs could be integrated into human tumor and neural organoids, leading to the development of hierarchically organized, structurally robust blood vessel networks.^105^ Importantly, these networks exhibited dynamic growth, responsiveness to angiogenic factors such as VEGF, and, crucially, successful anastomosis with the host vasculature upon transplantation. This functional integration highlights the potential of MPC-driven vascularization for creating organoids with enhanced physiological relevance and translational potential.

MSCs are particularly valuable due to their multipotency, robust secretion of pro-angiogenic and trophic factors, and capacity to differentiate into mural lineages.^101^ Mural cells are indispensable for vessel assembly, stability, and maturation. Pericytes, with their intimate coverage of endothelial tubes, promote vessel maturation, regulate hemodynamic forces, and release factors such as angiopoietin-1 for vessel quiescence.^106^ Mural cells offer structural and mechanical support reminiscent of native vessels, contributing essential signaling cues (e.g., PDGF-BB, TGF-β, Notch ligands) and ECM components (e.g., laminins, collagens) for vessel formation and integrity. Crucially, mural cell dysfunction underpins pathologies where the blood-brain barrier (BBB) is compromised (e.g., Alzheimer’s disease, seizures, stroke), making them vital for accurate disease modeling.^107^^,^^108^^,^^109^ Beyond direct lineage contributions, MSCs promote angiogenesis in diverse contexts. Summer et al. demonstrated that co-cultured MSCs and HUVECs formed interconnected networks within avascular human platelet lysate matrices.^110^ Intriguingly, MSCs can also indirectly mediate pro-angiogenic effects by modulating how ECs and other cells respond to factors such as tropoelastin.^111^

The influence of macrophages on angiogenesis makes their integration essential for accurate modeling of pathologies such as cancer, atherosclerosis, and those triggered by microbial infections. In co-culture with ECs, macrophages polarized toward a pro-inflammatory phenotype can dramatically enhance endothelial sprout length and density, likely through the modulation of Notch signaling pathways.^103^^,^^112^ However, recent studies suggest a broader role for diverse macrophage phenotypes in vascularization. Moore et al. demonstrated that encapsulating ECs specifically with M2 and M0 macrophages in a bioactive hydrogel significantly promoted vascular tubule formation, while M1 macrophages did not.^113^^,^^114^ Earlier work by Spiller et al. expanded this view, indicating both M1 and M2 macrophages contribute to angiogenesis, but likely fulfill distinct roles in the complex processes of vascular development, remodeling, and neovascularization.^115^ The intricate interplay between macrophages and tissue-specific vascular cells remains an active research frontier. Nonetheless, incorporating macrophages into vascularized organoids necessitates careful consideration, as their plasticity and responsiveness to biomaterial cues can introduce additional complexity into these models.

An additional strategy for vascularizing organoids involves the use of isolated microvessel fragments.^116^ This technique involves the meticulous harvesting of intact microvessels, preserving their basement membrane, associated pericytes, and SMCs, from source tissues such as adipose. These fragments are subsequently embedded within developing organoids, providing a pre-formed, rudimentary vascular network. Studies employing microvessel fragments in MSC-derived and adipocyte organoids have demonstrated rapid vascularization, with new vessels sprouting from the embedded fragments and anastomosing with the nascent organoid vasculature within days, ultimately establishing perfusable networks.^99^^,^^116^ Notably, this strategy significantly enhanced adipocyte differentiation and lipolytic function within the adipocyte organoids. Key challenges with this approach include ensuring the long-term viability of the incorporated microvessels and optimizing the microvessel source to maximize compatibility with the specific organoid system. Emerging solutions focus on the development of advanced bioengineered scaffolds tailored to promote microvessel fragment survival and seamless integration. Moreover, ongoing research prioritizes identifying ideal microvessel isolation sources that closely align with the organoid being modeled, potentially reducing immune mismatch responses.

The generation of physiologically relevant engineered vascularized tissues and organoids necessitates the strategic provision of organotypic signaling cues—derived either from the complex biochemical composition of the organ-specific microenvironment or directly from resident organ-specific vascular cells. Lippmann et al. pioneered the induction of BBB-like properties in hiPSC-derived ECs through retinoic acid exposure and co-culture with neural progenitor-derived astrocytes and pericytes, highlighting the power of this multi-lineage approach.^117^ Subsequent refinements yielded a cost-effective method for generating human brain microvascular ECs (BMECs) using Essential 6 (E6) medium,^118^ later optimized by Pong et al.^119^ specifically for BMEC expansion and cryopreservation. Alternative BMEC generation strategies include differentiation from hPSC-derived EPCs or immortalized cell lines such as BC1.^120^ Intriguingly, targeted activation of specific signaling pathways (e.g., Wnt, Notch) with small molecules offers a complementary, potentially more chemically defined approach for recapitulating BMEC development from hPSCs.^121^

Despite their widespread use, it is crucial to recognize the inherent limitations of using only HUVECs when striving for high-fidelity modeling of tissue-specific vascular niches. ECs exhibit remarkable structural and functional heterogeneity across organs.^122^ This diversity is shaped by the intricate interplay between their microenvironment, differential responses to growth factors (e.g., VEGF and FGF isoforms), and organ-specific regulatory mechanisms.^123^ Recent advances in single-cell RNA sequencing (scRNA-seq) have provided unprecedented insights into this heterogeneity. Marcu et al. characterized ECs from the developing human heart, lung, liver, and kidneys, revealing distinct transcriptomic signatures and specialized functions tailored to each organ.^124^ These findings underscore the importance of utilizing organ-specific ECs to advance organ regeneration, accurately model diseases with vascular components, and refine hPSC differentiation protocols aimed at generating endothelium with precise phenotypic and functional characteristics.

Co-culture systems offer a potent means to promote the expression of tissue-specific markers and accelerate the maturation of vascularized *in vitro* systems. In their seminal study, Takebe et al. successfully vascularized liver buds by combining human iPSC-derived hepatic endoderm cells, MSCs, and HUVECs.^125^ This triculture system fostered the spontaneous formation of vascular-like structures within the liver buds.^125^ While these vessels were not characterized for the expression for liver sinusoidal EC (LSEC) markers, their functionality was apparent in the enhanced liver bud maturation. Sato et al. extended this concept, generating a vascularized human placenta from iPSC-derived organ bud transplants.^126^ Crucially, co-culture systems can rapidly fine-tune endothelial differentiation. Helle et al. demonstrated that, within a mere 48 h of co-culture with iPSC-derived cardiomyocytes, iPSC-derived ECs adopted characteristics of cardiac-specific ECs, exhibiting increased maturity and homogeneity.^127^ Correspondingly, the inclusion of LSECs in liver organoids significantly enhances their structural and functional maturation.^128^^,^^129^ Yap et al. elegantly demonstrated the development of vascularized hepatobiliary organoids through the co-culture of liver progenitor cells (LPCs) with LSECs.^128^ Notably, LPC/LSEC organoids displayed robust hepatocyte-like cell formation, biliary duct development, and dramatic upregulation of hepatic and biliary genes within 7 days—features conspicuously absent in LPC-only organoids, underscoring the power of this approach.^128^

### Co-culture with vascular organoids

The strategy of co-culturing lineage-specific organoids (e.g., brain, heart, liver) with vascular organoids exploits the intrinsic capacity of ECs to undergo neovessel formation in response to pro-angiogenic signals from surrounding tissues. Researchers may establish co-cultures at early stages of organoid development or embed pre-formed vascular organoids into maturing organoids.^125^^,^^130^ The optimal timing depends on the desired outcomes and the specific organoid system, as early co-culture may promote deeper vascular penetration, while the use of pre-formed vascular organoids could expedite functional perfusion. Vascular integration typically proceeds through defined stages. ECs within vascular organoids sprout and migrate toward the target organoid, followed by anastomosis of the nascent vessels. Ultimately, a perfusable vascular network is established within the organoid. Importantly, timelines vary depending on the complexity of the organoid being modeled but can range from days to weeks.^131^ Sun et al. used this novel strategy to create vascularized brain organoids by integrating brain-specific vascular organoids with cerebral organoids.^132^ Remarkably, this resulted in the inclusion of microglia that respond to immune stimuli. This approach enables the study of cerebrovascular characteristics and the interactions between neuronal and non-neuronal elements in brain development and functioning.

While significant progress has been made, several challenges remain in optimizing organoid vascularization. A central need is to develop culture conditions that fully support the growth and differentiation of both the organoid and its associated vascular organoids. Research is ongoing to tailor biomaterials and ECM compositions that promote targeted vascularization in a tissue-specific manner.^133^ There is an unmet need for hydrogels that optimize organoid-specific vascularization. This is in part due to the limited understanding of the diversity of mature ECs and their developmental needs. Controlling the extent and patterning of vascularization within organoids is another critical area for refinement. As is discussed in subsequent sections, OOC microfluidic platforms and organoid 3D bioprinting offer potential to recapitulate the organotypic vascular architecture seen in native tissues. Further research into the molecular and genetic regulation of ECs could enable precise tuning of their responsiveness to organoid-derived cues for controlled vascularization.

### Organoid co-differentiation

Co-differentiation strategies for organoid vascularization center on inducing hPSCs toward vascular lineages concurrently with inducing various proportions of mesoderm, endoderm, and ectoderm to produce organ-specific lineages.^134^^,^^135^ By strategically modulating the Wnt/β-catenin, Activin/Nodal, and SMAD pathways, researchers coax hPSCs toward the mesodermal lineage alone or concurrently with the ectodermal and endodermal lineages to produce lineage-specific cardiac, neural, and hepatic organoids, for example, with integrated vascular cells. Key growth factors and small molecules such as VEGF-A, BMP4, FGF-2, angiopoietins 1 and 2, SB431542, PDGF-BB, and TGF-β are orchestrated in a temporally precise manner to promote EC and smooth muscle cell proliferation, migration, and vascular network assembly. Crucially, the timing and sequential exposure to these factors are meticulously controlled to mimic the dynamic signaling environment of *in vivo* development. Moreover, the maturation status, cell-type ratios, and long-term maintenance of vascular structures must be considered. This approach mirrors natural developmental processes, potentially yielding more physiologically relevant vascular networks. It fosters integrated development of organ-specific and vascular cell types within the lineage-specific organoid, making it particularly valuable for studying vascularization mechanisms within the context of specific organs.

Geometric factors such as colony size, shape, density, and spacing are pivotal in pluripotent stem cell maintenance and differentiation.^136^ Moreover, studies have demonstrated that the geometric confinement of hPSC colonies can result in self-organized patterning.^137^^,^^138^^,^^139^ Yamanaka and colleagues recently provided insights into how tight-junction complexes potentially shape morphological patterning during gastrulation in *in vitro* hiPSCs.^140^ Building on these findings, Abilez et al. developed an *in vitro* model, recapitulating the initial stages of human cardiac vascularization seen during the first 3 weeks of *in vivo* development.^135^ By utilizing spatially micropatterned hPSCs to form 2D gastruloids, the researchers generated cardiac vascularized organoids (cVOs), achieved using a single-pot approach following mesodermal induction. By day 16, the presence of cardiomyocytes, ECs, endocardial cells, SMCs, pericytes, epicardial cells, fibroblasts, and precursor cells was confirmed. The introduction of a specific combination of growth factors to the micropatterned hiPSCs and intermediate gastruloids led to the formation of a robust, perfusable, and branched vascular network within the cVOs. Furthermore, the team applied a similar co-differentiation method to generate vascularized hepatic organoids (hVOs). Following mesoendodermal induction, hepatic cells, SMCs, and ECs were formed by day 20. Such co-differentiation methods, as demonstrated by the cVO and hVO models, may suggest an emerging approach for vascularizing organoids.

Rather than solely relying on the growth factor-based differentiation of stem cells, direct reprogramming offers an alternative route to generating ECs from existing cell populations within a developing organoid. During development, intricate networks of transcription factors work in concert to orchestrate the complex process of cell specification. Hence, transcription factor overexpression in pluripotent stem cells or developing organoids can be used to specifically drive EC fate and promote vascularization. Lee et al. successfully converted human postnatal dermal fibroblasts directly into mature ECs using the transcription factor ER71/ETV2, bypassing the need for stem or progenitor stages.^141^ This approach yielded reprogrammed ECs capable of maturing and incorporating into vascular networks. Thus, ETV2 has been described as a master switch. More recently, Palikuqi et al. demonstrated that transient ETV2 reactivation in mature human ECs, combined with culture in a serum-free 3D matrix, “resets” these cells to a vasculogenic state.^142^ This reset enables the cells to self-assemble into perfusable, multilayered vascular networks within microfluidic chambers. ETV2 acts by remodeling chromatin and activating the RAP1 pathway, promoting the formation of complex vascular structures.

Overexpressing endothelial regulators (ETV2, FLI, ERG, KLF2) in hPSCs therefore offers a powerful way to prime them for differentiation into the endothelial lineage. Viral transduction and CRISPR-based editing deliver and activate key transcription factors, inducing a cell fate switch—reprogramming cells to adopt an endothelial identity. Cakir et al. introduced a method to induce vascular-like networks within human cortical organoids by ectopically expressing ETV2 in hESCs, resulting in improved functional maturation and BBB characteristics.^143^ The researchers demonstrated that ETV2 expression triggers EC differentiation from hESCs across various conditions (EB, neuron, and EC differentiation) without relying on traditional growth factors such as VEGF. This finding underscores the potent role of ETV2 in inducing EC formation irrespective of the differentiation pathway. Nonetheless, the degree of vascularization was low, and the organoids lacked perivascular cells, such as SMCs. Dailamy et al. subsequently developed an innovative method to enhance organoid vascularization by combining directed differentiation with genetic overexpression.^144^ Using this approach, the researchers created neurovascular and myovascular organoids by inducing the overexpression of *NEUROD1* (neural) and *MYOD1* plus *BAF60C* (muscle), respectively, in developing organoids. They observed complete vascular networks, including critical lineages such as SMCs and MSCs. in addition, the organoid constructs integrated tissue-specific parenchymal cells, which were lacking earlier vascular organoid models.

While a core set of transcription factors governs endothelial development, there exists a high degree of organ-specific specialization. Weiss and colleagues developed a method to generate complex organoids from hiPSCs by using a transient pulse of *GATA6* expression to trigger co-differentiation of all three germ layers. This approach yielded organoids with a liver bud-like phenotype, containing diverse cell types including hepatocytes, ECs, and stromal cells.^145^ Importantly, these organoids exhibited functional maturity, producing liver-specific proteins at levels comparable to human hepatocytes. Moreover, a different group has shown that the overexpression of *PROX1* and *ATF5* alongside targeted *CYP3A4* activation significantly enhances the vascular networks and improves the functionality of human liver organoids.^146^ Finally, Skylar-Scott et al. developed a technique known as orthogonally induced differentiation (OID) to differentiate hiPSCs into multiple lineages simultaneously by overexpressing specific transcription factors, circumventing the constraints of traditional differentiation media.^147^ They showed the utility of OID by producing vascularized cortical organoids from hiPSCs co-differentiated into ECs and neurons. Moreover, combining OID with multi-material bioprinting, they successfully fabricated patterned neural tissues. The identification of differentiation transcription factors and regulatory networks specific to different organs remains an area of active research.

### Organoid-on-a-chip (OOC)

Despite significant progress in developing complex organoid model systems, achieving functional vascularization often remains reliant upon transplantation into host animals.^125^^,^^148^ The inherent limitations of *in vivo* transplantation present a bottleneck for larger-scale applications. These constraints include high costs, ethical considerations, potential immune incompatibility between host and graft, and inter-animal variability that may obscure results. Consequently, there is an urgent need to develop sophisticated *in vitro* platforms capable of independently supporting robust vascularization and maturation of organoids. Such platforms would offer a scalable, precisely controlled, and ethically sound alternative to *in vivo* models, accelerating research in disease modeling, drug discovery, personalized medicine, and regenerative therapies.

Within the last decade, OOC platforms have emerged as a powerful solution. These microfluidic systems leverage precise spatial and temporal control to promote the vascularization and enhanced maturation of organoids (Table 2). OOC technology integrates organoid cultures into dynamic microenvironments that closely simulate *in vivo* conditions. Importantly, the fusion of OOC and organ-on-a-chip technologies creates a synergistic platform for developing physiologically complex *in vitro* models, offering enhanced translational potential.^149^

**Table 2:** OOC platforms for different tissue and organ types

OOC	Sources	Application	Scaffold	Microfluidic chip fabrication	Key findings	Vessel characteristics	Reference
Brain	hiPSCs	BEM and microfluidic device to improve the structural and functional maturation of human brain organoid	BEM, Matrigel	technique: soft lithography materials: PDMS solution and Sylgard 184 (10:1 ratio) molding: 2.2 mm on patterned master holes: 8-mm diameter, punched bonding: oxygen plasma assembly: stacked PDMS layers with bottom seal sterilization: autoclaved, UV dried flow: rocker system D, 8 mm; H, 0.33 mm; W, 0.9 mm	• BEM has 352 proteins known to show elevated expression in the human brain, while Matrigel has only nine such proteins • BEM-microfluidics organoids were more physiologically relevant and larger than Matrigel organoids (1.84 mm vs. 1.56 mm), with some reaching 4–5 mm • larger populations of RGCs along the VZ in BEM organoid • BEM promoted cortical layer development (day 45) • BEM organoids can be used to model several neurological disorders, providing a balance between cell-type diversity and consistency in organoid growth	N/A	^150^
	hiPSCs, MCF-7	investigating the impact of exosomes derived from breast cancer cells on brain neurodevelopment	Matrigel	material: PDMS technique: soft lithography structure: two layers; bottom with 1 mm micropillars, top with a 24-well plate ring process: mix PDMS and curing agent (10:1), degas, cure at 80°C (40–60 min), peel off mold	• brain organoids treated with exosomes showed an increased population of *OCT4*+ cells across multiple days of exposure. The exosomes potentially impaired neurodevelopment of brain organoids	N/A	^151^
Intestine	HUVECs, mouse ISCs	perfusable mini-gut tubes from stem cells that mimic the intestine’s structure and functions	collagen I, Matrigel	compartments: central hydrogel chamber for organoid culture. Basal side reservoirs for medium diffusion. Inlet/outlet reservoirs for perfusion design features: phase-guiding with semi-walls and pillars. Extra port for hydrogel loading fabrication: designed with CleWin, patterned on silica molded with SU8 photoresist, then PDMS. Plasma-treated PDMS bonded to glass dishes sterilization: cleaned, UV sterilized, and stored sterile	• rapid establishment of a confluent cell sheet in tubular hydrogel scaffolds colonized with mouse ISCs • larger than 3D organoids • cellular diversity closely resembles *in vivo* conditions and contains cell types rare or absent in traditional organoids • perfusable epithelial tissues were formed	N/A	^152^
	hiPSCs and HIMECs the epithelial cells were derived from duodenal organoids	–	collagen I, Matrigel	method: Photolithography and demolding of cured PDMS from a master mold PDMS ratio: 15:1 (prepolymer to curing agent) dimensions: cell culture channel (1 × 10 × × 0.15 mm), vacuum chambers (1.68 × 9.09 × × 0.15 mm), wall thickness 100 μm porous membrane: made by casting PDMS over micropatterned silicon, then overlaid and cured with a silanized PDMS slab assembly: bonded with corona plasma treatment; vacuum chambers formed by removing membrane sections bonding: final assembly cured at 80°C for permanent bonding tubing: silicone tubing with connectors for medium and suction flow: monolayer formation achieved with physiological fluid flow (60 μL/h) chip activation: intestine chip subjected to peristalsis-like motions mechanical stimulation: 10% strain at 0.2 Hz, applied via cyclic suction to side chambers	• successful perfusion and mechanical deformation, mimicking peristalsis • mechanical cues improved differentiation and formation of well-polarized epithelium with high density of distinct villus-like structures (∼30/cm^2^) • cultures that included HIMECs achieved epithelial confluence faster (2 days) compared to those without endothelium (6 days) • HIMECs played a pivotal role in quick epithelial confluence and possibly barrier function • EC-lined microchannels modeled drug absorption, bioavailability, and contributions of circulating immune cells	• perfusable vessels	^153^
Kidney	H9 hESCs, hiPSCs, hGMECs, HUVECs	culturing kidney organoids under millifluidic conditions	gelatin-fibrin	ink: two-part silicone elastomer, 10:1 ratio, homogenized 3D printing: custom perfusion gaskets, using a bioprinter with a 410-μm nozzle features: gaskets on glass, organoid chamber (15 × 3.6 × × 60 mm), 1 mm ECM organoids: space for 4–20 per chip, in 8 × 3.6 × × 20-mm area curing: 80°C, then autoclaved FSS range: low FSS (1 × 10^−7^ to 1 × 10^−4^ dyn/cm^2^), High FSS (8 × 10^−3^ to 3.5 × 10^−2^ dyn/cm^2^)	• the presence of perfusable lumens supported by mural cells • application of flow significantly enhanced organoid maturation • formation of more refined glomerular and tubular structures • improved nephron segment specification and functionality due to the fluid shear stress (∼1 dyne/cm^2^) provided by the millifluidic system	• uneven perfusion within the vessels (100-nm beads)	^154^
Liver	HepaRG, HUVECs, monocyte-derived macrophages, LX-2 (stellate cell line)	–	PET	material: COC - TOPAS from microfluidic ChipShop perfusion: silicone tubing for oxygen chip body dimensions: 75.5 mm (L) × 22.5 mm (W) × 1.5 mm (H) upper channel dimensions: 15.0 mm (L) × 2 mm (W) × 0.45 mm (H) lower channel dimensions: 16.8 mm (L) × 2 mm (W) × 0.40 mm (H) membrane dimensions: 13 mm (L) × 8.5 mm (W) × 0.02 mm (H), with 8-μm pore diameter membrane distances: to upper sealing foil: 0.7 mm to lower sealing foil: 0.8 mm flow rates and shear stress: upper channel: 50 μL/min, shear stress: 0.7 (dyn∗s)/cm^2^ lower channel: 1 μL/min, shear stress: 0.01 (dyn∗s)/cm^2^	• hepatic and vascular cell layers were grown on opposite sides of a suspended microporous membrane, which modeled the space of Disse • perfusion only on the vascular side • the model recapitulated oxygen gradient mimicking *in vivo* conditions and contained all major liver cell types. HepaRG cells dynamically adapted to normoxic and hypoxic conditions	• formation of highly confluent EC layer	^155^
	HepG2/C3A	liver-on-a-chip platform for long-term culture of 3D human liver spheroids	PMMA, PDMS	multilayer chips: PDMS-membrane-PDMS sandwich structure for spheroid culture; uses PET microporous membrane (3 μm pores, 2 × 10^6^ pores/cm^2^) for observation upper fluidic layer: designed in AutoCAD, made with soft lithography (2,000 × 200 μm channels) from SU8-2075 on silicon, using PDMS (10:1) lower microwell layer: CNC-milled PMMA master creates 1,080 microwells, converted into a smooth PDMS mold via a secondary PDMS-coating technique, then final PDMS molding (10:1) cured at 80°C for smooth concave microwells	• spheroid-based 3D liver-on-a-chip • microporous membranes modeled the fenestrated ECs in the liver • hepatic spheroids cultured in shallow concave microwells under high mass transfer and low shear stress conditions • minimal spheroid loss under perfusion conditions • higher expression of cytochrome P450, urea, and albumin relative to conventional 3D perfusion models (day 12)	N/A	^156^
Lung	lung cancer tissue (surgical resection)	culturing 3D lung cancer organoids and conducting drug sensitivity tests within a single system	Matrigel	3D culture methods: includes hanging-drop, biopolymer encapsulation, perfusion bioreactors, and cell sheet layering MPS platform: PDMS-based microfluidic channels for streamlined cell seeding and drug testing design: 29-well device with wells 750 μm deep and 500 μm wide flow: organoids mixed with Matrigel and medium, centrifugally loaded into wells, with a yarn capillary to regulate flow at 2–5 mL/day	• microfluidic platform (29 wells, 750-μm depth, and 500-μm width) cultured LCOs under physiological flow conditions and delivered specific drug concentrations to the LCOs via diffusion • LCOs (200 μm) morphologically resembled typical SCLC lesions • increased expression of stemness markers (*CD133*, *SOX2*, and *NANOG*) under perfusion conditions compared to static Matrigel droplet conditions	N/A	^157^
	A549, HUVECs, NHLFs	a microphysiological system to model lung cancer by combining 3D tumor spheroids with a self-assembled, perfusable microvasculature	fibrin	fabrication: used soft lithography for PDMS medium reservoir and channel slab dimensions: culture chamber 1,600 × 400 μm, microchannels 400 × 400 μm, reservoir 12 × 12 × × 4 mm process: mixed PDMS with curing agent (10:1), cured at 65°C, added ports, assembled with spin-coated PDMS, and re-cured ECM coating: incubated microchannels with fibronectin solution (25 μg/mL) for 3 h at 37°C channel washing: washed once with EGM-2 cell seeding: introduced 10-μL HUVEC suspension (1 × 10^7^ cells/mL) into channels, allowed attachment for 3 h perfusion setup: connected external reservoirs and syringe pump, set flow rate to 70 μL/h	• 3D organotypic model of vascularized human lung adenocarcinoma used for drug (paclitaxel) screening and toxicity assessments • the platform featured a cell culture chamber, an open top, and parallel microchannels for perfusion • the tumor spheroids were mixed vascular cells in fibrin hydrogel supplemented with aprotinin • clinical dose of paclitaxel resulted in endothelial apoptosis, oxidative stress, and vascular inflammation	• 3D networks of interconnected endothelial tubes, forming perfusable vessels • the engineered vessels anastomosed with endothelialized side channels • vessel formation was based on self-assembly of ECs and fibroblasts, with vessels exhibiting average diameter of 24 ± 7.05 μm (mean ± SD)	^158^
Neurovascular	hiPSC-derived ECs, pericytes, and neuroepithelial cells	co-culture of vascular cells and cerebral organoids on a 3D printed microfluidic chip	Matrigel	perfusion: connected to Chemyx pump, perfused at 2 μL/min solution: 1 μm RF-BEADS (1:1,000), fluorescein-40-kDa dextran (500 μg/mL) in PBS visualization: epifluorescence and confocal microscopy for beads and dextran	• cerebral organoids were seeded on day 5 and vascular cells on day 6 • enhanced penetration of ECs in the co-culture condition relative to mono-brain organoids • increased maturation rate of neurons (*NeuN*+ cells) at days 15 and 30 in the co-culture condition relative to mono-brain organoids • reduction in mature neuron markers in co-cultures compared to mono-brain cultures	• high expressions of endothelial and pericyte markers and formation of intact, lumenized vessels (>1 μm) • invasion of Matrigel (80 μm/day), leading to formation of complex vascular structures	^159^
Pancreas	hiPSCs	islet-on-a-chip model generated from heterogeneous hiPSC-derived islet organoids	Matrigel	design: multilayer microfluidic chip for islet organoid generation composition: top and bottom PDMS layers, through-hole PDMS membrane, polycarbonate porous membrane function: 3D culture of EBs, media perfusion, interconnected flow between upper and bottom channels flow: continuous culture medium was injected at 100 μL/h advantage: circulatory flow for efficient medium exchange and uniform fluid stress on organoids	• islet organoids comprise heterogeneous islet-specific α and β-like cells, showing enhanced expression of pancreatic β-cell specific genes and proteins • the platform supported the maintenance of organoid morphology (spherical shape with smooth edges) compared to control • islet organoids in the chip were more physiologically relevant, mimicking mature β-cells and had robust response to glucose	N/A	^160^
Placenta	primary EVTs, ECs, stromal cells, and uNK cells (endometrial biopsies)	implantation-on-a-chip to mimic the 3D organization of the maternal-fetal interface and model the invasion of EVT into the uterus and spiral artery remodeling during implantation	collagen, Matrigel	Fabrication: Soft lithography with PDMS on an SU-8 master for microchannels. design model is a 3D microfluidic device consisting of three parallel lanes: ECM, simulating specialized maternal endometrium; vascular chamber consisting of human uterine ECs, simulating maternal spiral artery; fetal section consisting of human EVTs assembly: sealed with a PDMS layer; top layer includes 7-mm media reservoir holes sterilization: UV irradiated for 20 min surface prep: poly(dopamine) coating for ECM hydrogel attachment, then rinsed and dried	• without trophoblasts, the maternal endothelium showed low apoptosis • the introduction of EVTs led to significant activation of apoptotic pathways in ECs • notable rise in caspase-3-positive ECs and per-cell caspase expression post-EVT invasion, aligning with physiological spiral artery remodeling processes	• EVT invasion disrupts the endothelium, leading to disorganization and reduced VE-cadherin expression, compromising vascular integrity	^161^
Prostate	LNCaP, PC3	PCa-MPS model to recapitulate epithelial features of PCa and CRPC cells and their PSA and miRNA secretion	agarose, collagen I	chip used: HUMIMIC Chip2 MPS (TissUse, Berlin, Germany) cells cultured: LNCaP and PC3 under dynamic conditions setup: two gels per perfusion circuit in the chip’s culture chambers media: 250 μL per chamber, perfused at 1 Hz for 4 days analysis: supernatant and cell samples collected from conventional, 3D static, and dynamic MPS cultures	• LNCaP cells formed spheroids, influenced by hydrogel density • MPS enhanced cytoskeletal, adhesion protein, and cancer marker expression, indicating an intensified cancer phenotype • MPS reduced PSA secretion and expression in LNCaP cells, suggesting dynamic culture impacts PSA levels • fluidic conditions affected androgen-sensitive and -insensitive cells differently, with implications for cell growth and PSA expression • cytoskeletal response was enhanced in LNCaP under MPS, linking to PSA expression changes	N/A	^162^
Retina	hiPSC (RPE and RO)	a retina-on-a-chip model that mimics human retinal functions and interactions, aiming to advance drug testing and research into retinal diseases	hyaluronic acid	platform: microfluidic for hiPSC-derived RPE and RO culture with physiological structure configuration: four micro-tissues linked by microchannel, in two-layered biocompatible polymers layers: top for RO/RPE compartments, bottom for nutrient perfusion barrier: porous membrane for nutrient exchange, protects from shear forces procedure: seed RPE cells, culture 24 h inject ROs in hyaluronic hydrogel to separate from RPE culture: initiated for 3 days, stable up to 21 days for analysis or further experiments	• the model integrated over seven types of retinal cells from hiPSCs, providing a comprehensive model • it achieved vasculature-like perfusion and the interaction between photoreceptors and RPE • enabled the formation of outer segment-like structures and in vivo-like functions such as phagocytosis and calcium dynamics • the platform successfully reproduced retinopathic side effects of chloroquine and gentamicin, showcasing its utility in evaluating ocular toxicity • it is a promising avenue for retinal disease study and therapeutic development	N/A	^163^
	hESCs (H9 and CSC14)	development and validation of a shear stress-free micro-millifluidic bioreactor to standardize and automate the maintenance of retinal organoids	Matrigel	design: SolidWorks-created mold with 200-μm channels and 2-mm chambers in a 6 × 5 array for RtOg culture, compatible with 96-well plates 3D printing: Formlabs form 3B, clear resin; post-processed with isopropanol, air-dried, UV cured fabrication: PDMS cast in 10:1 ratio, degassed, cured at room temperature over the mold assembly: PDMS demolded, ports punched, air plasma-treated, bonded to a glass coverslip	• RtOgs were cultured on a shear stress-free microfluidic bioreactor (31–37 days) • no observable morphological differences between static and bioreactor cultured RtOgs • less oxidative stress (LLS) signatures in bioreactor cultured RtOgs • the micro-millifluidic bioreactor supported long-term culture of RtOgs in a shear stress-free environment	N/A	^164^
Vascular	NC8 (hiPSCs), HUVECs	a microfluidic platform to cultivate and vascularize 3D cell aggregates	collagen I-Matrigel	device material: COC for durability, mass production, optical clarity, and chemical stability chip design: 10 microchannels, monitored with a 10-channel syringe pump encapsulation method: adapted hydrodynamic trapping for precise organoid placement within serpentine-shaped microchannels organoid positioning: fibrin hydrogel-embedded organoids accurately located at trap sites, maintaining morphology trap dimensions: adjustable based on organoid size; e.g., BVOs (Ø 600 μm, width 300 μm, height 800 μm), spheroids (Ø 300 μm, width 200 μm, height 400 μm)	• HUVEC networks formed functional anastomosis with BVOs • perfused hierarchical vascular network observed under flow • direct connections and functional vascular tree formation suitable for perfusable organoid studies were confirmed	• BVOs developed networks with ECs, pericytes, SMCs, basal membrane, hollow lumens, and tight-junction protein expression • direct connections between BVOs and HUVEC networks formed open microchannels. HUVECs showed 3D organization, CD31 positivity, and functionality confirmed by microbead perfusion • BVOs and HUVEC networks displayed a physiological hierarchy. Upstream/downstream HUVEC vessels were arteriole/venule sized (average 37 μm), internal BVO capillaries averaged 8 μm, and were surrounded by pericytes expressing SM22	^165^

BEM, brain ECM; CRPC, castration-resistant prostate cancer; BVO, blood vessel organoid; EVT, extravillous trophoblast; HIMECs, human intestinal microvascular ECs; LCO, lung cancer organoid; LNCaP, lymph node metastatic cancer prostate cell line; ISCs, intestinal stem cells; uNK, uterine natural killer; RtOg, retinal organoid.

OOCs feature microfluidic devices that provide controllable and dynamic microenvironments to promote the formation of functional vascular networks in organoids. They can be fabricated using soft lithography, where elastomers such as polydimethylsiloxane (PDMS) are cast onto microfabricated molds and subsequently bonded to a substrate, forming sealed microchannels.^166^ The widths of microchannels are exceedingly narrow, resulting in high surface-to-volume ratios and altered intermolecular forces.^167^ Fluids are controlled at the microscale, ranging from microliters to picoliters.^167^ Hence, the resultant fluid dynamics are significantly different from the macroscale. Fluid flow is predominantly laminar, characterized by a low Reynolds number, resulting in predictable linear flow patterns in which layers slide past each other with minimal mixing.^168^ Crucially, the laminar flow ensures efficient nutrient and oxygen supply, closely emulating *in vivo* conditions.^168^ It also allows for the establishment of stable and controlled angiogenic gradients. Furthermore, microfluidic platforms can be engineered to enable the co-cultures of ECs and supporting mural cells, promoting vessel maturation and integrity. Due to the high surface-to-volume ratios within microchannels, the effects of capillary action and surface tension are significantly enhanced.^167^ As such, many microfluidic systems utilize passive pumping and fluid control within the channels, obviating the need for external forces to drive flow.^167^ Nonetheless, it is essential to simulate *in vivo* interstitial flow patterns and shear stresses within the microchannels, particularly for organoids that depend on fluid control for their maturation. Due to the predominance of laminar flow in OOC microfluidic devices, modeling of turbulent flow-related processes such as atherogenic inflammation of vessels has been lacking.

To address the critical need for perfusable vasculature within *in vitro* organoid models, Quintard et al. recently designed a novel microfluidic system enabling the formation of interconnected endothelial networks.^165^ Employing mesenchymal spheroids (aggregates of human fibroblasts and GFP-labeled HUVECs), they first established that dynamic fluid flow significantly promotes the self-assembly of these spheroids into vessel-like structures, as compared to static conditions. Subsequently, by integrating RFP-labeled HUVECs into a hydrogel matrix, a functional connection was established between a pre-existing endothelialized microchannel and a trapped mesenchymal spheroid. Remarkably, spontaneous anastomosis occurred between these distinct EC populations, resulting in a continuous, interconnected network. Perfusion assays with fluorescent microbeads confirmed the functionality of the newly formed networks, demonstrating successful flow along the endothelial structures. As proof of concept, they showed anastomosis between HUVEC networks and blood vessel organoids. This seminal work demonstrates a powerful method for establishing functional vascularization within organoid systems, enabling the study of complex vascular processes in a controlled *in vitro* environment.

Despite the transformative potential of OOC platforms, certain limitations must be addressed to optimize their translational impact. Ensuring scalability and reproducibility of microfluidic devices is paramount. Batch variability stemming from fabrication processes can introduce inconsistencies in critical parameters influencing cellular behavior, undermining experimental outcomes. Refinement of fabrication techniques, along with rigorous quality control, is crucial. Integrating advanced biomaterials, diverse cell populations, and precise spatiotemporal control of biochemical cues will enhance the physiological relevance of OOC models. Furthermore, limitations associated with PDMS, particularly its absorption of small molecules, can hinder accurate pharmacokinetic studies. Exploring alternative materials, surface modifications, and incorporating computational modeling to mitigate PDMS interactions are essential for reliable drug screening applications. For instance, cyclic olefin copolymer (COC) was used by Quintard et al. for their chip fabrication for its minimal absorption of chemicals, optical properties desirable for imaging, and scalability.^165^

The utility of vascularized OOCs in preclinical modeling hinges on the functionality of their integrated vascular networks. Current assessments of these networks often rely on morphological metrics that offer limited insight into their oxygenation capacity. To address this shortcoming, Tronolone et al. recently developed a chained neural network trained on a diverse vascularized on-chip dataset, capturing variations in factors influencing vascular architecture.^169^ This network generated a vascular network quality index (VNQI) from diverse morphological inputs. VNQI demonstrated a significantly stronger correlation with experimentally measured oxygen levels within vascularized on-chip models than isolated morphological metrics. In a vascularized islet-chip subjected to hypoxia, VNQI positively correlated with transplantation success, highlighting its utility. This standardized approach to assessing vessel functionality promises to improve the predictive accuracy of vascularized on-chip models. In the future, artificial intelligence (AI) and machine learning (ML) could optimize vascularization of organoids by analyzing complex image data, identifying patterns predictive of optimal vessel formation. Moreover, algorithms could suggest modifications to fabrication techniques or culture conditions to enhance vascularization based on predicted outcomes.

Finally, the future of OOC looks promising, especially given the shift toward integrating multiple organoid types (microphysiological systems) on a single chip, leading to the development of body-on-a-chip systems. Diverse tissue types can be co-cultured in these complex systems, each residing in dedicated chambers but interconnected through a shared microfluidic circulation. Edington et al. integrated up to 10 different microphysiological systems through a vascularized network and evaluated organ-organ interactions, drug metabolism, and systemic responses.^170^ More recently, Vunjak-Novakovic and colleagues developed a patient-specific multi-organ chip from mature tissues and used the system for therapeutic testing and mechanistic studies. The tissues were separated by a selectively permeable endothelial barrier, providing functional insights into tissue-specific endothelium and possible endothelial plasticity.^171^ These body-on-a-chip models represent a significant step toward achieving personalized drug testing. The convergence of ML and *in silico* modeling with microfluidic systems will facilitate real-time monitoring and predictive analysis, enhancing the potential of these platforms in translational research.^172^

### Organoid 3D bioprinting

The generation of functional vascular networks remains a fundamental obstacle in tissue and organoid engineering. To ensure post-implantation viability, constructs must possess perfusable vasculature that seamlessly integrates with the host circulatory system. Conventional *de novo* vascularization approaches struggle to achieve this, often producing vessels lacking functionality or proper anastomotic connections. Additionally, the stochastic organization of these networks significantly hinders efficient perfusion, jeopardizing engineered tissue survival and function. Bioprinting has arisen as a transformative technique, demonstrating the potential to overcome these limitations through the controlled fabrication of perfusable vascular networks. It surpasses traditional approaches by enabling the precise, layer-by-layer deposition of biomaterials, hydrogels, and cells—a methodology conducive to constructing intricate vascular patterns.^173^^,^^174^ This technique’s potential extends far beyond simple vascularization; it offers the opportunity to address the organ donor crisis through the eventual development of customized, transplantable organs. Naturally, hurdles persist—fully replicating the hierarchical complexity of native vasculature and ensuring long-term graft functionality necessitates further research and optimization.

In some instances, a prerequisite for successful vascular bioprinting is the acquisition of detailed 3D representations of the target vascular architecture.^54^^,^^175^ This necessitates the use of sophisticated vascular imaging modalities. Micro-computed tomography (micro-CT), with its high-resolution capabilities, is particularly well suited for delineating small-caliber vessels.^176^^,^^177^ Magnetic resonance angiography (MRA), while offering slightly lower resolution, excels due to its non-invasive nature and capacity to image deep tissues without exogenous contrast.^178^^,^^179^ For real-time visualization of fine vascular structures, optical coherence tomography (OCT) provides unparalleled micrometer-level resolution.^180^^,^^181^ Techniques such as confocal microscopy and ultrasound-based imaging, although not providing the same high-resolution detail, offer additional data valuable for bioprinting workflows. Importantly, the data gleaned from these modalities must be incorporated into the bioprinting design and planning stages to ensure both anatomical fidelity and physiological relevance of the fabricated vascular networks.

Bioinks, the cell-laden hydrogels that serve as the fundamental building blocks in bioprinting, play a pivotal role in determining the success of vascularization within engineered tissues. To facilitate the complex cellular interactions essential for the formation of perfusable, mature vascular networks, next-generation bioinks require precisely tailored properties. Key areas for optimization include mechanical properties that mimic the native ECM, facilitating cell adhesion, migration, and vessel morphogenesis. Controlled degradation kinetics are crucial; the bioink must provide initial structural support, then degrade at a rate that aligns with neovessel formation and ECM deposition by embedded cells. Moreover, bioinks must promote favorable cell-biomaterial interactions, potentially through the incorporation of bioactive moieties that enhance EC attachment, proliferation, and the self-assembly processes required for functional vascular network generation. As previously discussed, recent innovations involve the exploration of decellularized ECM-based bioinks, synthetic materials with tunable viscoelasticity, and hybrid bioinks that combine natural and synthetic polymers.

Several primary bioprinting techniques for vascularization are highlighted in Table 3, including extrusion-based bioprinting (EBB), which is optimal for printing large-scale (∼centimeter) structures^182^; laser-assisted bioprinting (LAB), recognized for its high resolution (∼micrometer)^183^; and inkjet-based bioprinting (IBB) (∼tens of micrometers), which provides rapid printing speed.^184^ Each technique has undergone modifications for better reproducibility and to accommodate various bioinks. The choice of bioprinting method significantly influences the vasculature’s structure, functionality, and complexity in engineered organoids, underscoring the ongoing need for technological refinements.^185^ Additionally, the ability to create vasculature from the micrometer to the centimeter scale theoretically allows the modeling of both laminar- and turbulent-flow physiology and pathophysiology, which is a limitation of current OOC platforms discussed above.

**Table 3:** General organoid 3D bioprinting techniques and their approaches to organoid vascularization

Bioprinting technique	Vascularization methods	Advantages	Limitations
EBB	• bioink is deposited through a nozzle under pneumatic or mechanical pressure • multi-material extrusion can be used to print sacrificial inks that are later removed to create vascular channels • these channels can then be lined with ECs to form blood vessels	• ability to print cell-laden hydrogels • suitable for high cell densities • multi-material printing	• relatively low resolution • possible shear stress on cells during extrusion • prone to clogging • slower printing speeds
LAB	• a laser pulse is focused on a ribbon coated with bioink, leading to high-pressure bubble generation that propels droplets of bioink onto a collector substrate • cells are precisely deposited in a pattern, allowing for the formation of vascular-like structures via induction	• high precision and spatial resolution • no nozzle clogging • minimal shear stress on cells	• requires complex equipment • expensive
IBB	• Thermal or piezoelectric actuation is used to eject bioink droplets from a nozzle. • layer-by-layer deposition, often using supporting scaffolds. Vascular structures can be formed through self-assembly or directed maturation	• fast printing speed • relatively inexpensive • scalable for large-scale production	• can be limited to low-viscosity materials, restricting organoid complexity • resolution may be lower than LAB

Indirect 3D bioprinting offers a versatile approach for generating complex and perfusable vascular networks within engineered organoids. This strategy relies on temporary, sacrificial materials integrated within the organoid matrix.^186^ These materials, often including biodegradable polymers or fugitive inks, are subsequently removed to create hollow channels that strategically mimic natural vascular structures. Sacrificial materials must be carefully selected based on their biocompatibility, ease of dissolution, and structural properties. Pluronic F127, with its reversible thermogelation properties, has been widely used for this purpose.^182^^,^^187^ However, its potential for cytotoxicity at higher concentrations (10% w/w) warrants careful consideration.^188^

Sacrificial bioprinting, a sub-category of indirect 3D bioprinting, finds particular relevance in organoid vascularization. Here, fugitive bioinks are printed into the organoid construct and later removed, leaving behind channels that are subsequently endothelialized. This controlled process offers advantages over relying solely on EC self-assembly, enabling the precise design of functional vascular networks.^149^ Miller et al. demonstrated this concept using 3D-printed carbohydrate glass as a sacrificial template to engineer organoid-like constructs with perfusable, endothelialized vascular networks.^149^ Kolesky et al. further advanced this approach by integrating sacrificial bioprinting to fabricate organoids with embedded vasculature, multiple cell types, and ECM.^182^ Utilizing materials such as PDMS and Pluronic F127, they achieved notable progress. However, limitations in perfusion highlighted the need for further optimization. Subsequently, Kolesky et al. refined their multi-material bioprinting approach, enabling the creation of thick (>1 cm) vascularized human tissues suitable for sustained perfusion, a significant milestone for organoid engineering.^189^

Sacrificial writing into functional tissue (SWIFT) offers another powerful indirect bioprinting strategy for organoid vascularization. Developed by Skylar-Scott et al., SWIFT leverages organ building blocks (OBBs) that possess self-healing and viscoplastic properties.^190^ The densely packed OBBs serve as the organoid matrix, while printed sacrificial materials, often gelatin based, form the vascular network template. Upon removal of the sacrificial material, the resulting microchannels are endothelialized. The precision of SWIFT allows fabrication of complex vascular architectures within organoids, as demonstrated by Skylar-Scott et al., who created a perfusable cardiac organoid exhibiting synchronous beating. Despite significant advancements, indirect 3D bioprinting and sacrificial templating strategies still face challenges in achieving the capillary-level resolution (5–10 μm) required for optimal organoid function.^63^ Innovations in bioink design and high-resolution printing modalities are needed to address this limitation and create a more seamless interface between bioprinted vessels and the organoid microenvironment. Furthermore, ensuring the long-term stability and integration of these vascular networks within dynamic organoid systems remains a critical area for continued research.

Direct 3D bioprinting offers distinct advantages for organoid vascularization by enabling the simultaneous deposition of cells and the biomaterials that define the vascular architecture. This integrated approach streamlines the fabrication of vascularized organoids, providing exceptional control over spatial cell placement and vascular patterning. Such precision is paramount for recapitulating the complex and essential interactions between organoid tissues and their supporting vasculature. EBB is a widely utilized direct bioprinting modality in organoid vascularization. EBB’s adaptability to multiple materials and variable compositions is particularly beneficial when working with the diverse cell types often found within organoids. Crucially, bioink rheology must be carefully optimized to ensure both cell viability and the formation of stable tubular structures that will effectively integrate with the organoid.^191^^,^^192^

Coaxial bioprinting further enhances vascularization strategies for organoids by enabling the creation of multilayered or simple tubular vascular structures. This precision is essential when replicating the intricate architecture of organoid microvasculature. The use of naturally derived biomaterials such as alginate, collagen, GelMA, and chitosan promotes favorable cellular interactions within the organoid microenvironment.^193^ By optimizing parameters such as nozzle configuration, bioink viscosity, and extrusion rate, coaxial bioprinting can successfully generate vessels tailored to the specific diffusion and perfusion requirements of individual organoid types.^193^^,^^194^^,^^195^^,^^196^

Freeform reversible embedding of suspended hydrogels (FRESH) bioprinting presents a transformative solution to the challenge of fabricating mechanically weak, yet biomimetic, vascular structures within organoids. By extruding bioinks directly into a supportive thermoreversible gel bath, FRESH enables the high-fidelity construction of complex, cell-laden vascular channels.^197^^,^^198^ This technique is especially valuable for organoids that require intricate vasculature for optimal nutrient and waste exchange. The integration of FRESH with coaxial bioprinting, as demonstrated by Gao et al. in their atherosclerosis model, highlights the potential for creating exceptionally realistic organoid vascular networks.^199^ Importantly, the choice of bath material, such as vessel-derived extracellular matrix (VdECM), can further enhance biocompatibility and physiological relevance.

ECM patterning strategies offer an alternative and sometimes complementary approach to bioprinting for organoid vascularization. These techniques leverage the intrinsic properties of ECM materials to guide cellular self-assembly and vessel formation. Bischel et al. first used a technique known as viscous finger pattering (VFP) to efficiently pattern lumens within type I collagen hydrogels in microchannels.^200^ They showed the application of VFP in generating diverse channel geometries and multiple hydrogel layers. While offering the potential for spontaneous vascular network generation, ECM patterning may introduce limitations in terms of precise control over vascular architecture when compared to direct bioprinting methods. Continued advancements in direct bioprinting, particularly in the areas of bioink development and high-resolution printing modalities, promise to further enhance vascularization strategies for organoids. The integration of these techniques with advanced imaging modalities will enable the design and fabrication of organoid-specific vascular networks that seamlessly integrate with the native cellular microenvironment, promoting long-term organoid viability and functionality.

## Future outlook

The advancements made in organoid vascularization demonstrate the revolutionary potential of bioengineering methods. Within this exciting context, we are in an era that promises to be even more transformative. The incorporation of multiple vascular beds in organoids is essential for future advancements. Current methods have predominantly focused on modeling arterioles, capillaries, and venules. Integrating the lymphatic system could provide a more accurate representation of *in vivo* conditions and enhance organoid functionality. In addition, developing organoid models that seamlessly integrate the vascular and immune systems will help elucidate complex cellular interactions during inflammation and disease progression. Furthermore, given the recent growth of personalized medicine, developing vascularized organoids tailored to individual patients is an attractive prospect. This is particularly important in modeling patient-specific drug interactions and uncovering the mechanisms of hereditary diseases.

As we anticipate technological progress in this decade, the next generation of bioinks is expected to include materials with superior biocompatibility and functional integration. Hence, organoids with enhanced stability, longevity, and function could be generated by combining these enhanced bioinks with advanced bioprinting techniques, including 4D bioprinting. Additionally, given the sheer volume of data generated from organoid studies, it is essential to integrate advanced bioinformatics and ML more fully with stem cell biology. This will potentially provide optimal vascularization protocols, using predictive and generative models to refine bioengineering methods.

As discussed in this review, several challenges hinder the translation of lab-scale vascularized organoid prototypes to clinically and commercially viable constructs. The key translational barriers include issues with reproducibility, scalability, and cost-effectiveness. The intricate cellular composition of organoids and the complex conditions under which they are developed lead to batch-to-batch variability. This lack of reproducibility can limit organoids' physiological relevance and reliability in therapeutic applications. Scaling up the production of organoids from the academic research environment to meet commercial needs requires advanced high-throughput technologies. Considering the complexities of vascularizing these constructs, it can be difficult to ensure uniformity and consistency in nutrient supply and waste removal and maintain sterility across larger production volumes. However, without scalability, the promise of organoids in drug discovery and regenerative therapies remains unrealized. Furthermore, the production of vascularized organoids can be cost-prohibitive. This is partly due to the costs associated with specialized equipment, advanced bioinks, growth factors, and small molecules. Without cost-effective production, the scalability and, thus, accessibility of organoid-based therapies will be necessarily limited. Addressing these pressing translational barriers is necessary for the broader integration of vascularized organoids in clinical and research landscapes. Moreover, market and economic insights must be integrated throughout the development process. Similarly, clear guidelines around patent rights, intellectual property, and potential commercial interests are necessary.

Finally, the rapid advances in organoid technology inexorably raise important practical and ethical questions. These questions broadly concern the derivation, procurement, and use of human stem cells in research and therapeutic settings. Transparent and ethical sourcing of stem cells should remain a high priority, and the rights of patients and donors must be upheld. In addition, there are ethical and regulatory questions specific to the potential use of organoids in regenerative therapies. Foremost among them is the concern around safety and efficacy. We need to fully understand how vascularized organoids behave once transplanted, especially regarding the potential for tumor formation. Furthermore, as vascularization methods improve, it is reasonable to speculate about the potential for brain organoids to advance in size and complexity beyond current limitations. As such, at what juncture does the complexity of an organoid raise new ethical questions? Moreover, as technological advancements lag behind equity, particularly in the setting of developing countries, is there a risk that organoid-based therapies and transplantable organs become restricted to wealthy nations? We are confident that the answers to these open questions will become apparent as the field progresses in the coming years.
